# Tumor suppressor genes and KRAS G12C inhibitor resistance in non-small cell lung cancer

**DOI:** 10.1016/j.isci.2025.114429

**Published:** 2025-12-12

**Authors:** Linsha Zhu, Xiangbo Jia, Lei Xu, Li Chen, Xiangning Fu, Hua Yan, Bo Ai, Shu Peng

**Affiliations:** 1 Department of Thoracic Surgery, Tongji Hospital, Tongji Medical College, Huazhong University of Science and Technology, Wuhan, P.R. China; 2 Department of Thoracic Surgery, Henan Provincial People’s Hospital, People’s Hospital of Zhengzhou University, School of clinical medicine, Henan University, Zhengzhou, Henan, P.R. China; 3 Department of Breast Surgical Oncology, National Cancer Center/National Clinical Research Center for Cancer/Cancer Hospital, Chinese Academy of Medical Sciences and Peking Union Medical College, Beijing 100021, P.R. China

**Keywords:** Therapeutics, Gene network, Cancer

## Abstract

KRAS G12C inhibitors (G12Cis) have revolutionized the treatment of cancers driven by this historically undruggable mutation, offering unprecedented clinical responses in non-small cell lung cancer (NSCLC) and other malignancies. However, both primary and acquired resistance rapidly curtail their efficacy. Emerging clinical and preclinical evidence underscores the heterogeneity of resistance mechanisms. Strikingly, in KRAS-driven NSCLC, a common phenomenon is co-mutations in tumor suppressor genes (TSGs), which orchestrate resistance through multifaceted pathways such as sustained proliferation, metabolic reprogramming, phenotypic plasticity, and immune microenvironment remodeling. Accordingly, this review summarizes relevant reasons underlying diverse resistant mechanisms in KRAS G12C-mutated NSCLC, with an emphasis on deciphering the mechanism of tumor suppressor gene (TSG) alterations serving as key mediators linking oncogenic KRAS dependency to therapeutic resistance. Our research continued to discuss relevant preclinical models to facilitate the advancement of the study of these resistance mechanisms.

## Graphical abstract

**Figure:**
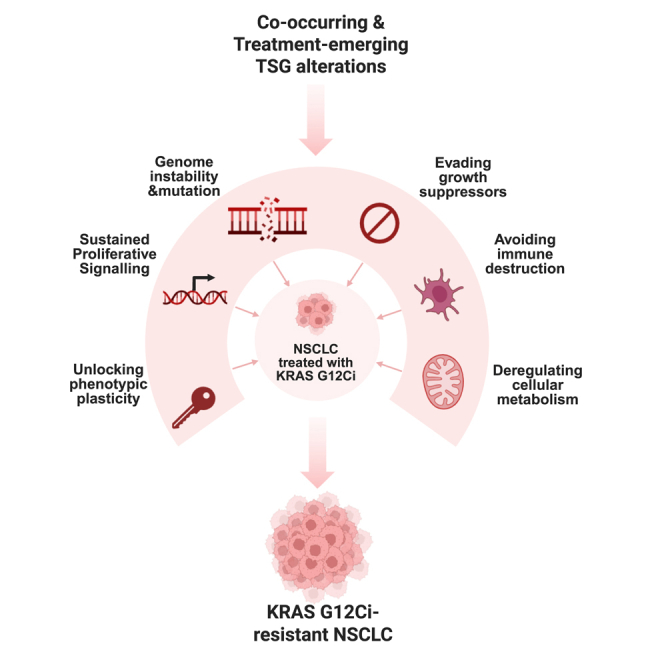

## Introduction

KRAS mutations occur in approximately 30% of non-small cell lung cancer (NSCLC), with the G12C subtype accounting for about 40% of these cases.^1^^,^^2^^,^^3^^,^^4^^,^^5^ This mutation results in a glycine-to-cysteine substitution at codon 12, impairing GTPase-activating protein (GAP)-mediated GTP hydrolysis and locking KRAS in its active GTP-bound state. Consequently, downstream pathways such as RAF-MEK-ERK, PI3K-AKT-mTOR, and RALGDS are constitutively activated, driving tumorigenesis.^6^^,^^7^

For decades, the prognosis for KRAS-mutated patients has been a serious issue. In NSCLC, KRAS G12C was nearly mutually exclusive (≤1.2%) with all actionable driver mutations (e.g., ALK rearrangements, EGFR mutations, and ROS1 rearrangements), limiting targeted treatment options.^8^^,^^9^ Currently, immunotherapy and chemotherapy are the main approaches for KRAS-mutant patients; although some KRAS G12C cases can derive clinical benefit, both treatments lack specificity.^5^^,^^10^^,^^11^^,^^12^^,^^13^^,^^14^ Early attempts to target KRAS signaling indirectly, through membrane localization or downstream effectors, yielded limited clinical success.^15^ Moreover, KRAS was long considered “undruggable” due to its picomolar affinity for GTP, high intracellular GTP concentrations, and the absence of deep hydrophobic pockets for drug binding.^16^^,^^17^

A breakthrough came with the discovery of covalent KRAS G12C inhibitors (G12Cis), which exploit the mutant cysteine to bind a cryptic allosteric pocket adjacent to the switch II region, trapping KRAS in its inactive GDP-bound state.^18^ This strategy capitalizes on the intrinsic nucleotide cycling of KRAS G12C, which exhibits faster GTP hydrolysis than other KRAS mutants, increasing the pool of GDP-bound target available for inhibition.^19^^,^^20^^,^^21^ Sotorasib (AMG 510) and Adagrasib (MRTX849) were the first two G12Ci inhibitors to demonstrate clinically meaningful efficacy.^22^^,^^23^ Several next-generation G12Ci (e.g., divarasib, LY3537982) have also shown promising preliminary activity in early-phase trials.^24^

Despite these advances, the clinical efficacy of G12Ci has not matched that of other targeted agents such as EGFR tyrosine kinase inhibitors.^25^^,^^26^^,^^27^^,^^28^ Both primary and acquired resistance represent major clinical challenges. Importantly, in NSCLC, the compendium of co-occurring genomic alterations, particularly tumor suppressor gene (TSGs) inactivation, produces a more significant impact on tumor heterogeneity than oncogenic driver mutations alone.^29^ Frequently co-mutated TSGs in KRAS-mutated NSCLC include serine/threonine kinase 11 (*STK11)/liver kinase B1 (LKB1),* kelch-like ECH-associated protein 1 (KEAP1), tumor protein 53 (TP53), and cyclin-dependent kinase inhibitor 2A/B (*CDKN2A/B)*.^29^ Under KRAS G12Ci selective pressure, treatment-emerging TSG alterations such as retinoblastoma protein (RB), neurofibromatosis type 1 (*NF1),* or phosphatase and TENsin homolog deleted on chromosome 10 (PTEN) *can emerge*, further shaping resistance evolution.^30^^,^^31^ From an evolutionary perspective, these co-mutations suggest functional cooperation, rather than random accumulation. While oncogenic KRAS sustains proliferative and survival signaling, TSG loss amplifies these pathways and fosters adaptive resistance.

However, the mechanisms by which TSG alterations directly modulate G12Ci response remain incompletely defined. This review will primarily focus on the well-established resistance mechanisms elucidated from sotorasib and adagrasib, which currently constitute the bulk of the clinical and preclinical evidence. While newer G12Ci show encouraging activity, their resistance profiles are not yet fully characterized due to limited follow-up, and are therefore beyond our main scope. Herein, we synthesize the underlying causes of G12Ci resistance, with a particular emphasis on the multifaceted roles of TSGs, integrating insights from both clinical observations and foundational research.

## Clinical outcomes of KRAS G12Ci highlight resistance challenges

In clinical trials, both sotorasib and adagrasib revealed moderate efficacy and acceptable tolerability in patients with KRAS G12C-mutated NSCLC with prior treatments. In the phase 1/2 CodeBreaK100 trial, sotorasib achieved an objective response rate (ORR) of 41%, a median progression free survival (PFS) of 6.3 months, and a median overall survival (OS) of 12.5 months.^32^ However, in CodeBreaK 200 phase 3 trial comparing with docetaxel, sotorasib showed only a modest improvement in PFS (5.6 vs. 4.5 months) and no significant OS benefit, leading to FDA rejection for first-line use.^33^ Similarly, adagrasib demonstrated an ORR of 43% and a median PFS of 6.5 months in the KRYSTAL-1 trial, with its effect for earlier-line therapy under investigation in ongoing phase 3 trials.^34^ Collectively, there may be a high prevalence of primary resistance owing to the relatively low ORRs; the observed progression eventually in a phase 2 trial on sotorasib among all initial responders and no significant changes in OS, underscoring the emergence of adaptive resistance. The next-generation inhibitor divarasib has also reported promising activity, with an ORR of 53.4% and a median PFS of 13.1 months from a phase I study, yet resistance remains a challenge for all these agents.^35^ Subsequent clinical studies identified both primary and acquired resistance as major barriers to durable efficacy, with mechanisms including secondary mutations, bypass pathway activation, genomic determinants of primary resistance, and histologic transformation.^30^^,^^31^^,^^36^ Therefore, a clarification of relevant mechanisms is of great significance for optimizing therapeutic strategies and developing effective combination therapies.

## Underlying causes of rapid and diverse resistance mechanisms

Several causes may explain the rapid and diverse resistance mechanisms toward KRAS G12Ci.

Firstly, the inherent pharmacological profile of first-generation G12Ci presents a fundamental limitation. These agents, such as sotorasib and adagrasib, selectively target the inactive GDP-bound state of KRAS^G12C^, leaving a residual pool of GTP-bound KRAS^G12C^ capable of sustaining downstream signaling and fostering escape.^23^^,^^37^ The markedly superior efficacy of the next-generation inhibitor divarasib, which boasts ∼50-fold higher potency and deeper clinical responses, validates this model by demonstrating that more complete target inhibition translates to improved outcomes.^35^ This concept is further corroborated by the synergistic effect of SOS1 inhibitors such as MRTX0902, which suppress nucleotide exchange and shrink the active KRAS-GTP pool.^38^ Moreover, the non-uniform KRAS G12C nucleotide exchange rates within cancer cells may generate varied drug responses, whereas uniform silencing of KRAS via siRNA consistently induces a quiescent state, underscoring the role of target dynamics in shaping treatment heterogeneity.^39^

Secondly, the profound signaling plasticity of KRAS-mutant tumors facilitates a wide spectrum of bypass mechanisms. As a central signaling hub, KRAS inhibition disrupts feedback loops, triggering the heterogeneous reactivation of upstream receptor tyrosine kinases (RTKs), engagement of alternative RAS isoforms (NRAS/HRAS), or compensatory upregulation of KRAS, and exploitation of downstream pathway redundancy (MAPK, PI3K).^30^^,^^31^^,^^39^^,^^40^ This decentralized network architecture stands in sharp contrast to EGFR-mutant cancers, where resistance is often funneled through a narrower set of mechanisms.^41^

Thirdly, a permissive genomic landscape, often characterized by a high tumor mutational burden (TMB) linked to smoking history, accelerates the acquisition of diverse on-target secondary mutations.^4^^,^^5^^,^^42^ This is exemplified by the distinct and broader spectrum of resistance mutations (e.g., at G12, G13, Q61, R68, Y96, H95) observed in KRAS^G12C^-mutant NSCLC compared to the more uniform T790M-mediated resistance, which accounts for 50–60% of acquired resistance to EGFR-TKIs in EGFR-mutant lung cancer.^30^^,^^43^ Crucially, structural differences between inhibitors shape this mutational profile. Mutations at H95 (e.g., H95D/Q/R) confer resistance to adagrasib, but remain sensitive to sotorasib; while V8E, G13D, A59 S/T, and R68M mediate resistance to sotorasib, but exhibit sensitivity to adagrasib.^30^^,^^44^ The Y96D mutation, by altering the critical switch-II pocket, confers broad resistance to all first-generation G12Ci, even including the more potent divarasib.^45^ This underscores the feasibility of mutation-guided sequential therapy while highlighting the need for entirely new drug classes. In addition, high TMB also enhances the potential for the activation of alternate oncogenic signaling pathways aforementioned.^42^

Finally, and most critically for this review, the pre-existing biological heterogeneity of KRAS-mutant tumors, largely dictated by co-occurring genomic alterations—particularly in tumor suppressor genes (TSGs)—creates a fertile ground for diverse resistance mechanisms to emerge. This heterogeneity is evident in the spectrum of signaling dependencies: while some resistant models, like those derived from KRAS^LSL−G12C/+^/Lkb1^f/f (^KcL) mice, show downregulated KRAS signaling, indicating a shift away from oncogenic KRAS dependence,^46^ functional classification of KRAS-mutated cell lines further reveals a dichotomy between KRAS-dependent proliferation and KRAS-independent EMT-driven programs.^47^ Although different KRAS alleles (e.g., G12C vs. G12V) can exhibit distinct signaling biases,^48^^,^^49^ comprehensive multi-omics profiling has conclusively demonstrated that co-mutations in STK11, TP53, and CDKN2A/B, rather than the specific KRAS mutation subtype itself, serve as the principal architects of biological heterogeneity and therapeutic response in KRAS-driven cancers.^50^^,^^51^ This TSG-defined landscape may directly shape the resistance evolution in two major ways. First, it creates distinct cellular states that are intrinsically resilient. This is particularly evident in NSCLC, where non-genetic mechanisms, such as histologic transformation and EMT, are recognized as predominant resistance pathways, a finding underscored by the relatively lower frequency of acquired RAS/MAPK genetic alterations in NSCLC compared to CRC.^45^ Second, it creates a permissive background that actively influences the acquisition of genetic resistance mechanisms. For instance, TP53 inactivation, by promoting genomic instability, may increase the probability and rate at which secondary resistant mutations, including those at the drug-binding site, such as the G12C/Y96 double mutant, are acquired.

**Figure 1:**
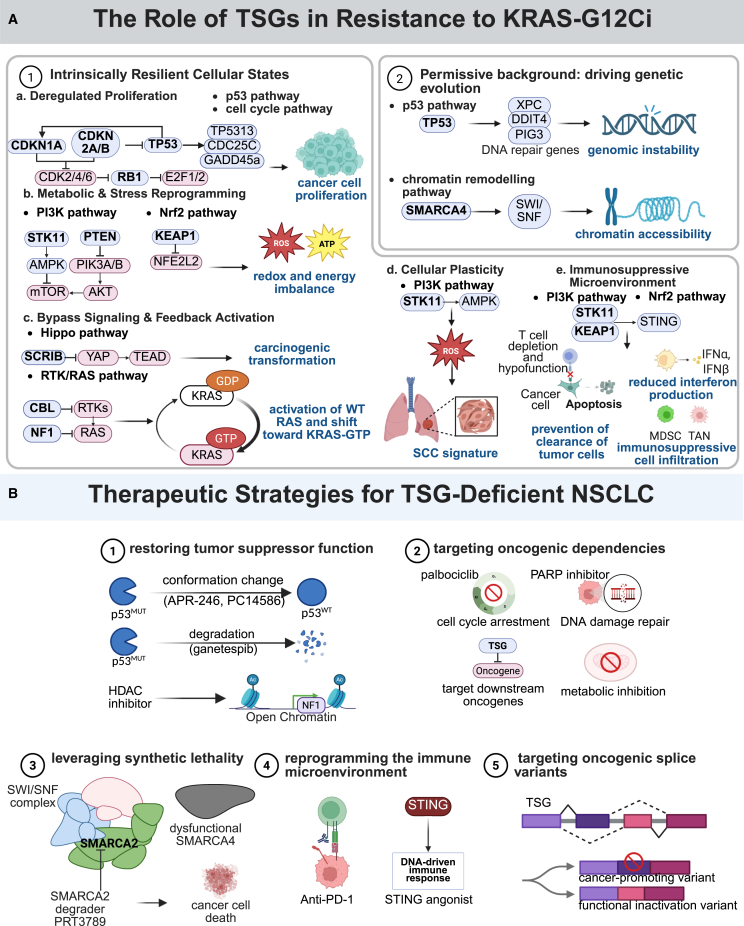
Conceptual framework of TSG-mediated resistance to KRAS G12Ci (A) This schematic illustrates the mechanisms by which the loss of tumor suppressor genes (TSGs) drives resistance to G12Ci. (B) This panel summarizes the core therapeutic approaches to target vulnerabilities arising from TSG loss.

At this stage, there is still a significantly insufficient elucidation of the precise role of TSGs, whether as pre-existing co-mutations or treatment-emerging alterations, despite advances in understanding KRAS G12Ci resistance mechanisms. Emerging evidence reveals that TSG alterations orchestrate resistance through multifaceted mechanisms, which may affect the immune microenvironment, disrupt proliferative signaling inhibition, deregulate cellular metabolism, promote epigenetic reprogramming, and enhance phenotypic plasticity, ultimately leading to heterogeneous intrinsic or acquired resistance in KRAS G12C-mutated tumors. The key TSGs and their associated pathways involved in resistance to KRAS G12Ci are summarized in Figure 1.

## Roles of tumor suppressor genes in resistance to KRAS G12Ci

### Serine/threonine kinase 11

STK11 is a critical TSG encoding a serine/threonine kinase that maintains cellular homeostasis through the activation of AMPK or suppression of mTOR signaling.^52^^,^^53^^,^^54^ Regarding KRAS, STK11 mutation is significantly enriched in KRAS G12C-mutated tumors compared to KRAS non-G12C-mutated tumors (20.59% vs. 5.95%, [OR] = 4.10) across all cancer types, indicating their potential relationship.^4^ This phenomenon, however, was not observed in lung cancer, possibly attributable to the co-enrichment of KRAS G12C and STK11 in NSCLC.^4^ The co-mutation frequency of KRAS G12C and STK11 was estimated to be around 23%.^55^ Ranks as the third most frequently mutated gene in lung adenocarcinoma and demonstrates a significant co-mutation propensity with KRAS mutations, collectively defining the distinct biological KL (KRAS/STK11 co-mutant subtype). This subtype exhibits enhanced tumorigenesis and metastatic potential due to the synergistic activation of downstream signaling pathways.^50^^,^^56^^,^^57^^,^^58^ The synergistic oncogenic effect between STK11 inactivation and KRAS activation has been revealed to arise from multifaceted factors involving epigenetic reprogramming, metabolic alterations, shifts in signaling pathway dependencies, and the shaping of an immunologically “cold” tumor microenvironment (TME), all of which have been deeply investigated previously.^29^^,^^59^

The impact of STK11 mutations on response to KRAS G12Ci remains controversial. For instance, a two-year analysis of the CodeBreak100 trial suggested potential long-term benefit for patients with STK11 mutations,^32^ whereas biomarker analyses from the KRYSTAL-1 trial reported a lower objective response rate (ORR) in this subgroup.^34^ Moreover, in the CodeBreak100 phase 2 clinical trial, a numerically higher response was observed in patients with STK11-mutant, KEAP1 wild-type (WT) tumors compared to other subgroups.^60^ A pooled analysis further concluded that STK11 mutations, without concurrent KEAP1 mutations, produced no significant impact on OS or ORR.^61^

The discrepancies in clinical observation strongly suggest a discordance between STK11 genotype and functional phenotypes. This mismatch is evident in the TCGA and OAK cohorts. A majority (77.6% and 44.3%) of tumors classified as “STK11-deficient” by transcriptional signatures retained the WT STK11 gene, whereas a small fraction (2.6–9.6%) of “STK11-proficient” tumors harbored STK11 mutations.^62^ For instance, a subset of STK11 WT tumors exhibited transcriptional signatures akin to STK11-deficient tumors.^57^ This paradox highlights two critical issues: diverse mechanisms of functional impairment and functional heterogeneity of the mutation itself. STK11/LKB1 functional loss can result not only from genetic mutations but also from epigenetic silencing, protein degradation, or non-coding RNA regulation.^52^^,^^63^ Structural studies also demonstrated that mutations such as Y49D, G135R, and D194Y disrupt the kinase domain and abrogate function, while a surface-exposed mutation such as R87K may retain kinase activity.^53^ Therefore, we conclude that the current binary classification of mutant and WT based solely on genetic sequencing is too crude to accurately capture the functional status of the STK11 pathway. Future biomarker development should pivot toward functional validation or functional annotation of mutations for better classification.

**Figure 2:**
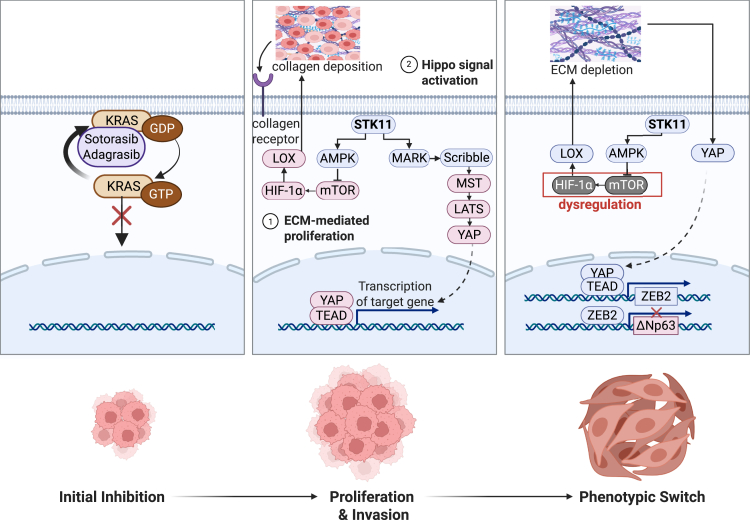
Adaptive evolution of STK11-deficient tumors under KRAS G12Ci in NSCLC Magenta indicates activated genes, while blue denotes inactivated genes. STK11 inactivation induces oxidative redox imbalance, driving upregulated mTOR-HIF-1α signaling that activates LOX to promote tumor proliferation. Excessive ROS accumulation due to failed compensatory responses may trigger AST as an adaptive resistance mechanism. This phenotypic switch is critically dependent on YAP inactivation (blue), which leads to the derepression of the key squamous driver ΔNp63. These opposing roles of YAP highlight the remarkable plasticity of STK11-null tumors in evading therapy.

In the context of STK11 loss, tumor cells exploit multiple mechanisms to evade KRAS G12Ci therapeutic pressure. LKB1 loss can activate the mTOR-Hif1α-LOX axis and catalyze ECM crosslinking, enhancing invasive and metastasis potential that may lay the groundwork for resistance.^54^^,^^64^ Beyond this, direct YAP/TAZ pathway activation constitutes a more immediate resistance hub. LKB1 was demonstrated to activate Hippo kinases.^65^ The drug resistance study conducted in KRAS-driven NSCLC cell lines with STK11 knockout or STK11 mutation revealed the activation of the YAP/TAZ pathway.^66^^,^^67^ Consistent YAP/TAZ activation mechanisms were also unveiled in G12Ci-resistant GEMMs as well as single-cell RNA sequencing of resistant human KRAS G12C tumors. However, a profound paradox emerges in adeno-to-squamous transition (AST). In KRAS-driven models, STK11 co-mutation uniquely predisposes to SCC differentiation.^68^^,^^69^ LKB1 loss dysregulates the mTOR-Hif1α axis, downregulating LOX and remodeling the extracellular matrix (ECM).^54^^,^^70^ This, in turn, inhibits Hippo/YAP signaling, leading to the derepression of the key squamous driver ΔNp63, ultimately driving AST, and is related to G12Ci resistance.^70^^,^^71^^,^^72^ Clinical data from the KRYSTAL-1 trial validate this, showing that baseline SCC signatures correlate with diminished adagrasib efficacy specifically in the STK11-mutant subgroup; moreover, elevated expression of *KRT6A*, a marker of squamous differentiation, predicts poor prognosis in patients with adagrasib-treated NSCLC, and is significantly associated with co-occurring KEAP1 and STK11 mutations.^46^ These seemingly contradictory mechanisms—YAP activation versus inhibition, LOX upregulation versus downregulation—collectively illustrate the remarkable plasticity of STK11-null tumors. We propose a model of adaptive evolution: under initial stress, cells activate the mTOR-Hif1α-LOX axis to foster invasiveness. When this proves insufficient against gradually increasing pressure, a phenotypic switch to squamous differentiation is initiated, necessitating LOX downregulation, ECM remodeling, and YAP inactivation to unlock the AST program via ΔNp63. Future work must delineate the molecular cues that dictate the choice between these adaptive pathways. Figure 2 summarizes the potential mechanisms of STK11 in KRAS G12Ci resistance.

Beyond cell-autonomous resistance mechanisms, the STK11 mutation-driven immunosuppressive tumor microenvironment (TME) may also compromise the efficacy of G12Ci. As while agents such as AMG 510 can promote a pro-inflammatory, T cell infiltrated TME to synergize with immunotherapy.^22^ STK11 mutations intrinsically drive a profoundly immunosuppressive.^12^^,^^73^ Clinical evidence from rapid-autopsy studies of KRAS G12Ci-resistant patients has revealed parallel immune evasion patterns, including reduced neoantigen burden, diminished lymphoid signatures, and expanded mast cell populations.^74^ Mechanistically, STK11 loss orchestrates this state through dual metabolic and epigenetic reprogramming: it upregulates MCT-4 to enhance lactate efflux, which engages GPR81 on immune cells to promote M2 polarization and T cell dysfunction,^75^ while concurrently silencing the STING pathway via EZH2/DNMT1-mediated hypermethylation to blunt interferon production and antigen presentation.^62^^,^^76^^,^^77^ The resultant “cold” TME, marked by CD8^+^ T cell depletion and functional impairment,^78^^,^^79^ creates a permissive niche for therapy resistance. Therefore, further elucidation is still necessitated to get into the bottom of the precise interplay among metabolic reprogramming, epigenetic silencing, and immune cell crosstalk, thereby developing effective combinatorial strategies.

### Kelch-like ECH-associated protein 1

KEAP1 is a critical repressor of the NRF2 transcription factor. Under normal conditions, KEAP1 binds NRF2, promoting its ubiquitination and degradation. Under oxidative stress or carcinogen exposure, in contrast, KEAP1 undergoes structural change, releasing NRF2 to activate antioxidant and detoxification genes, eventually amplifying cellular defense mechanisms.^80^ However, KEAP1 LOF mutations or inactivation may disrupt this regulation, leading to NRF2 accumulation and sustained activation of pathways supporting cancer cell survival, including antioxidant synthesis, xenobiotic metabolism, and drug efflux.^81^ KEAP1 loss was found to exacerbate tumor burden and accelerate progression to high-grade lesions in a conditional Kras^LSL−G12D/+^/Trp53^f/f^ mouse model, highlighting its role in both tumor initiation and progression.^82^ Clinically, KEAP1 has been reported to be associated with poor efficacy toward immunotherapy, radiation, chemotherapy, and targeted therapy.^8^^,^^78^^,^^83^^,^^84^ In addition, KEAP1 mutations would drive KRAS G12Ci resistance via NRF2-mediated glutaminolysis and oxidative stress buffering in KRAS-driven NSCLC.^82^

According to estimation, KEAP1 exhibited a mutation frequency of approximately 10%–15% in NSCLC^59^^,^^85^ and up to 20% in KRAS-mutated NSCLC.^5^^,^^81^^,^^83^^,^^86^ Across pan-cancer analyses, while KEAP1 mutations were enriched in KRAS G12C tumors (15.38% vs. 4.61% in non-G12C), this pattern was less pronounced in NSCLC-specific contexts, likely attributable to overlapping mutational landscapes and tissue-specific selection pressures.^4^^,^^5^ A key feature of KEAP1-mutant lung adenocarcinoma is its frequent co-occurrence with STK11 mutations. TCGA data indicate that KEAP1 alterations are present in 37.4% of STK11-deficient tumors, compared to only 1.3% in STK11 WT cases.^29^^,^^87^^,^^88^ Simultaneously, in the KL cluster, there was a significant accumulation of genetic events involving KEAP1 among both LKB1-mutation positive and negative LUACs.^50^ This co-mutation is facilitated by both chromosomal proximity (KEAP1, SMARCA4, and STK11 are clustered on chromosome 19p) and functional synergy in metabolic adaptation.^59^^,^^89^

**Figure 3:**
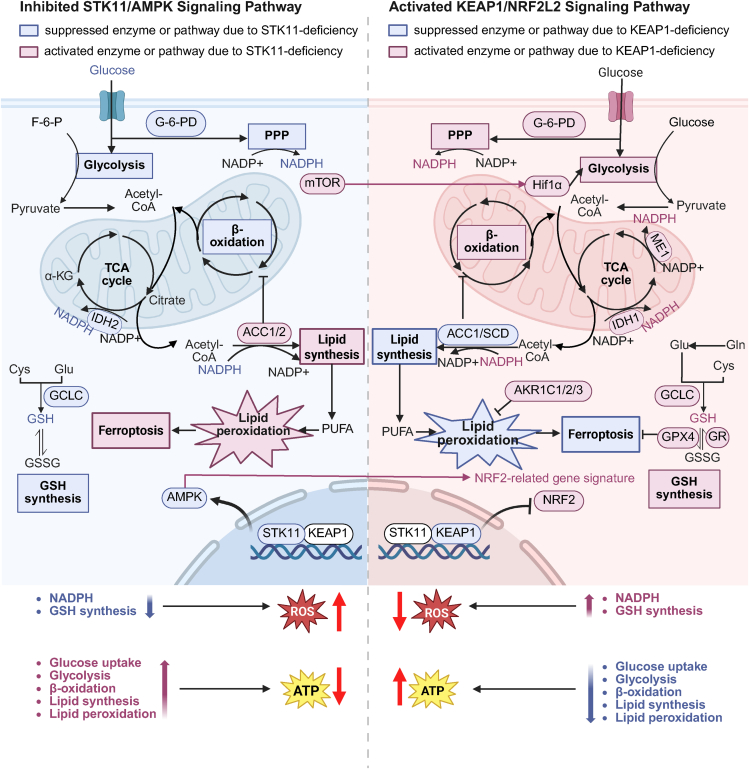
The crosstalk of STK11 and KEAP1/NRF2 promotes redox and energetic adaptation in NSCLC STK11 and KEAP1 exert opposing roles in glucose metabolism, GSH synthesis, lipid metabolism, and ferroptosis, with KEAP1 alterations partially rescuing STK11 loss-induced energetic and oxidative stress through NRF2-driven metabolic rewiring. Magenta indicates activated genes or biochemical processes, while blue denotes inactivated genes or biochemical processes. Abbreviations: F-6-P, fructose-6-phosphate; G-6-PD, glucose-6-phosphate dehydrogenase; PPP, pentose phosphate pathway; IDH2, isocitrate dehydrogenase 2; ME1, malic enzyme 1; Gln, glutamine; Glu, glutamate; Cys, cysteine; GCLC, glutamate-cysteine ligase catalytic subunit; GSH, glutathione; GSSG, glutathione disulfide; GPX4, glutathione peroxidase 4; GR, glutathione reductase; ACC1/2, acetyl-CoA carboxylase 1/2; SCD, stearoyl-CoA desaturase; PUFA, polyunsaturated fatty acid.

The functional STK11-KEAP1/NRF2 pathway interplay supports tumor survival through a coordinated metabolic rewiring (Figure 3). STK11 loss inactivates AMPK, impairing NADPH regeneration via fatty acid oxidation and creating redox stress.^90^ To survive, tumors upregulate the pentose phosphate pathway (PPP) via its rate-limiting enzyme glucose-6-phosphate dehydrogenase (G-6-PD), whose elevated expression has an established relationship with poor prognosis.^89^^,^^91^^,^^92^ This dependency was underscored by the sharp NADPH decline in KL tumors upon G-6-PD inhibition, a response absent in KP tumors.^89^ Concurrent AMPK suppression also limits glycolysis, increasing dependence on glutamine metabolism.^88^ KEAP1 loss further amplifies this adaptation through the NRF2-mediated activation of PPP flux, glutathione synthesis and glutamine utilization, collectively stabilizing redox and energy homeostasis.^57^^,^^88^ Notably, while STK11 loss alone sensitized cells to ferroptosis, KEAP1/STK11 co-mutations paradoxically conferred resistance via NRF2-driven antioxidant production and mTOR-Hif1α activation.^93^^,^^94^ Furthermore, oncogenic KRAS might exacerbate oxidative stress and anabolic metabolism, potentially synergizing with KEAP1/STK11 co-mutations to drive tumor aggressiveness.^95^

The adverse prognostic impact of KEAP1 co-mutations on KRAS G12C-mutated NSCLC has been documented by real-world studies.^96^^,^^97^ Patients with KEAP1 mutations experienced significantly worse PFS (2.8 vs. 5.4 months, *p* < 0.001, HR 2.26 [1.60–3.19]) and OS (6.3 vs. 11.1 months, *p* < 0.001, HR 2.03 [1.38–2.99]) on sotorasib.^39^^,^^61^^,^^98^ Moreover, patients harboring KEAP1 mutations exhibited lower ORR compared to KEAP1 WT counterparts across STK11 subgroups (STK11-mutant cohorts: 23% vs. 50%; and STK11 WT cohorts: 14% vs. 39%).^60^ This trend was subsequently corroborated by biomarker analyses from a phase 1–2 KRYSTAL-1 trial, where adagrasib achieved an ORR of 28.6% in KEAP1-mutant patients versus 51.7% in patients with KEAP1 WT.^34^ The lowest response (14.3%) was observed in patients with STK11 WT with concurrent KEAP1 mutations.^34^ This suggests that the concurrent loss of STK11 may, paradoxically, mitigate the strong negative impact of a solitary KEAP1 mutation. We hypothesize that this phenomenon may stem from underlying metabolic dysregulation. The loss of STK11 disrupts cellular energy homeostasis, which could partially offset the robust antioxidant and pro-survival activity conferred by KEAP1/NRF2 pathway activation. Supporting this hypothesis, re-expression of LKB1 in A549 cells (KRAS-mutated, LKB1-deficient, and KEAP1-inactivated) led to enhanced cell apoptosis under H_2_O_2_-induced oxidative stress.^88^ This interaction between KEAP1 and STK11 co-mutations, and its precise mechanistic link to therapy resistance, warrants further in-depth investigation.

Mechanistically, KEAP1 loss promotes KRAS G12Ci resistance through NRF2-mediated redox adaptation, although some evidence seems conflicting. KEAP1-NFE2L2 pathway enrichment was observed through single-cell RNA sequencing of resistant patient tumors, in turn supporting its role in adaptive resistance.^66^ Preclinical studies show that compared to KEAP1 WT H358 cells, HCC44 cells (KRAS G12C, and LKB1/KEAP1-mutated) exhibited marked resistance to ARS-1620, a phenotype attributed to KEAP1/NRF2-mediated redox buffering rather than LKB1 loss.^99^ Another study conducted CRISPR/CAS9 screening on H2122 cell lines (with p.G12C homo), and H2122 cell-derived xenografts, and revealed that KEAP1 loss would represent a mechanism of intrinsic or acquired resistance to MRTX849. But in their study, none of TP53, STK11, and KEAP1 mutations correlated with response or resistance in a series of cell line panels.^23^ Moreover, KEAP1 knockout using CRISPR/CAS9 in drug-sensitive H358 cells had only modest effects on the efficacy of both sotorasib and adagrasib when independently evaluated.^31^ Besides, KEAP1 mutations would also facilitate an immunosuppressive microenvironment, characterized by reduced T/B-cell infiltration, downregulated PD-L1, and suppressed STING signaling.^78^^,^^100^^,^^101^ These effects mirrored those of STK11 loss, creating a “cold” tumor milieu that synergized with metabolic reprogramming to limit therapeutic efficacy. Collectively, the interplay between KEAP1-driven redox adaptation, immune evasion, and metabolic dependencies underscores the need for combinatorial strategies to overcome resistance.

### Cyclin-dependent kinase inhibitor 2A/B

Cyclin-dependent kinase inhibitors, under cellular stress or DNA damage, have been found to be induced to halt cell cycle progression by inhibiting cyclin-dependent kinase, thereby suppressing the G1-to-S phase transition and preventing the replication of damaged DNA. The CDKN2A locus (9p21) encodes two tumor suppressors of p16^INK4a^ and p14^ARF^. Specifically, p16^INK4a^ can inhibit the activities of CDK4/6 to block RB phosphorylation and E2F-mediated transcription; while p14^ARF^ can stabilize p53 to enforce cell-cycle arrest or apoptosis.^102^^,^^103^The CDKN2B gene, adjacent to CDKN2A, producesp15^INK4b^, a CDK4/6 inhibitor that reinforces cell cycle control. In KRAS-mutated NSCLC, CDKN2A and CDKN2B were inactivated in around 20% and 12% of cases, respectively, with no significant enrichment in KRAS G12C versus non-G12C subtypes.^4^^,^^50^ Moreover, there was no significant difference in the co-mutation frequency of CDKN2A between KRAS G12C and non-G12C KRAS mutations.^4^ Additionally, the biallelic loss of CDKN2A/B defined the KC subgroup of KRAS-driven tumors, marked by RALGDS pathway dependency and invasive mucinous adenocarcinoma phenotypes linked to low NKX2-1 expression.^50^^,^^104^

Clinically, LOF CDKN2A/B is confirmed to be associated with inferior treatment efficacy toward KRAS G12Ci. In the CodeBreak 100 trial, two patients with NSCLC with baseline CDKN2A/B homozygous deletions and no treatment emerging alterations showed no response to sotorasib, but experienced disease progression,^31^ indicating primary resistance. In patients treated with sotorasib or adagrasib, co-alterations in CDKN2A were associated with worse PFS (3.4 vs. 5.3 months, *p* < 0.001, HR 1.98 [95% CI 1.32–2.97]) and OS (6.4 vs. 10.7 months, *p* = 0.009, HR 1.66 [95% CI 1.03–2.68]), though ORR was not significantly affected,^61^ suggesting CDKN2A as a contributor to rapid acquired resistance. Paradoxically, KRYSTAL-1 biomarker data showed a numerically higher ORR in CDKN2A-mutated patients (58.3% vs. 44.9%),^34^ possibly confounded by small sample sizes, coexisting mutations (e.g., KEAP1 or SMARCA4), or epigenetic mechanisms, considering that p16 promoter methylation (observed in 33% of NSCLCs) was synergistically enhanced by KRAS mutations in colon cancer and positively correlated with NSCLC.^105^^,^^106^^,^^107^ Notably, tumors harboring at least 2 co-mutations in KEAP1, SMARCA4, and CDKN2A displayed significantly worse outcomes on KRAS inhibitors, indicating the presence of additive resistance mechanisms.^61^ Another rapid autopsy study was carried out on a KRAS G12C-mutated lung adenocarcinoma patient who developed rapid resistance after initial response to AMG510, with the observation of downregulated G2/M checkpoint regulators and E2F target genes, among other overlapping resistance mechanisms.^74^ CDKN1A (encoding p21^CIP1/WAF1^), a p53-dependent pan-CDK inhibitor targeting CDK2/4/6, emerged as a potential sensitive marker in KRAS G12C models. CRISPR/Cas9 screening in H358 cells positioned CDKN1A on the positive side of the MAGeCK β score distribution, revealing that its activation might enhance sensitivity to ARS-1620.^39^

### Tumor protein 53

Generally, TP53 can suppress tumorigenesis by mediating oncogenic stress responses and activating target genes that regulate apoptosis, cell-cycle arrest, senescence, and DNA repair.^108^^,^^109^ For instance, TP53 mutations were highly prevalent in smoking-associated lung cancers, mirroring the smoking-related bias observed in KRAS mutations.^110^ It offers a reason to interpret the highest frequency of TP53 co-mutation in KRAS-driven NSCLC, occurring in approximately 50% of KRAS G12C-mutated tumors, a rate comparable to non-G12C KRAS and non-KRAS subtypes.^4^^,^^61^^,^^83^ While TP53 mutations primarily drove unchecked proliferation in KRAS-mutated cancers, co-mutations in STK11 or KEAP1 would impair immune surveillance.^56^ In addition, P53 alterations would remodel the tumor immune microenvironment, with the KP subgroup (KRAS/TP53 co-mutated) exhibiting concurrent enrichment of pro-immune and immune-evasion gene signatures.^47^^,^^111^

TP53 co-mutation status, despite being prevalent, lacks independent predictive value for KRAS G12Ci efficacy in clinical trials so far.^60^^,^^61^^,^^96^^,^^97^ This may stem from the functional heterogeneity of TP53 mutations, involving LOF, dominant-negative (DNE), and gain-of-function (GOF) variants.^112^^,^^113^ DNE mutants disrupted WT p53 activity to promote tumorigenesis,^112^ whereas GOF mutants conferred oncogenic properties indispensable for cell survival.^113^ The complexity of these functional alterations may collectively contribute to the absence of a significant impact of TP53 co-mutations on therapeutic outcomes. For instance, in EGFR-mutated NSCLC, nondisruptive TP53 mutations (associated with partial LOF and GOF) independently predicted shorter OS (17.8 vs. 28.4 months),^114^ highlighting the requirement for TP53 mutation stratification by functional class in KRAS-mutated contexts.

Notably, Runx3 is another tumor suppressor frequently deleted or silenced in KRAS-driven cancers,^115^^,^^116^ which can modulate the p14^ARF^-p53 axis, serving as an early defense against oncogenic KRAS. Runx3 inactivation was reported to disrupt this safeguard, enabling lung adenocarcinoma progression even without TP53 loss.^117^ This compensatory mechanism may obscure the contribution of TP53 mutations to tumor evolution in KRAS-mutated NSCLC, further complicating the prediction of therapeutic outcomes.

### PTEN

PTEN gene encodes a protein called PTEN, which functions as a TSG through its ability to dephosphorylate phosphatidylinositol 3,4,5-trisphosphate, thereby negatively regulating the PI3K/AKT pathway.^118^ According to prior research, PTEN inactivation might have greater clinical relevance in contributing to primary resistance in colorectal cancer treatment, given a rare co-occurrence of PTEN inactivation or PIK3CA activating mutations with KRAS mutations in lung cancer, in contrast to their 42.4% co-occurrence rate in colorectal cancer.^119^ Despite a frequent downregulation of protein expression in over 40% NSCLC, genetic alterations (e.g., deletions, insertions, and point mutations) occur only in approximately 2–7% of lung cancers.^120^ In clinical research, consideration at the protein level may further enhance our understanding of the impact of PTEN LOS on therapeutic efficacy.

Previously, in the CodeBreak 100 clinical trial on 43 patients undergoing sotorasib treatment, two patients with NSCLC were found to have treatment-associated PTEN mutations or deletion.^31^ In another KRYSTAL-2 study comparing pre-treatment and post-resistance samples from 38 patients, PTEN alterations were acquired in 2 patients, both of whom had colorectal cancer and exhibited additional alterations in the RTK/RAS/MAPK/PI3K pathway, suggesting the potential importance of PTEN LOF mutations in acquired resistance to adagrasib.^30^ Analysis of co-mutations in KRAS G12Ci-treated patients also revealed an enrichment of PTEN mutations among those experiencing early progression.^61^ However, these mutations were not significantly associated with the OS, possibly due to the high prevalence of KEAP1, SMARCA4, and CDKN2A mutations in the study population.^61^ In addition, knockdown of PTEN using shRNA conferred resistance to sotorasib in MIA PaCa-2 cells.^119^

### SMARCA4

SWI/SNF related BAF chromatin remodeling complex subunit ATPase 4 (SMARCA4) encodes BRG1, the catalytic ATPase subunit of the SWI/SNF chromatin remodeling complex, functioning significantly in regulating chromatin accessibility and gene expression. While its inactivation alone does not initiate lung adenocarcinoma, combined loss with p53 deletion and oncogenic KRAS activation (KPS model) drives more aggressive tumors than KRAS/p53 alone.^121^ SMARCA4 mutations, similar to KRAS mutations, are often smoking-associated and linked to high tumor mutation burden.^122^ In KRAS G12C-mutated NSCLC, SMARCA4 co-mutations occurred in approximately 10% of cases,^4^^,^^61^ and confer a clearly detrimental impact on KRAS G12Ci and immunotherapy outcomes. Among the two SMARCA4 mutation sub-categories, Class 1 mutations (truncations, fusions, or homozygous deletions) caused complete BRG1 loss and predicted poor OS, whereas Class 2 variants (missense mutations/variants of unknown significance) retained partial protein expression but still associated with adverse prognosis.^123^

At this stage, we know little about the dual role of SMARCA4 as both a tumor suppressor and context-dependent oncogene in KRAS-driven cancers.^121^^,^^124^^,^^125^ Cell lineage-specific effects have been documented in preclinical research, which indicated that club cell secretory protein (CCSP)-driven KRAS models exhibited accelerated tumorigenesis upon SMARCA4 loss, whereas surfactant protein C (SPC)-driven models showed suppressed progression.^126^ The hypothesis that SMARCA4-mutated tumors might primarily originate from club cell lineage might explain discrepancies within preclinical research or between preclinical and clinical studies.^126^ Class 2 missense mutations might further complicate this duality, potentially acting through GOF mechanisms, rather than simple LOF.^127^^,^^128^

Despite its context-dependent biological duality, SMARCA4 mutations exert a more pronounced negative effect on KRAS G12Ci efficacy and immunotherapy response compared to other frequently co-mutated tumor suppressors. Co-mutation analysis found that SMARCA4 co-mutations were associated with poorer PFS (1.6 vs. 5.4 months, log rank *p* < 0.001, MV HR 3.04 [95% CI 1.80 5.15]) and OS (4.9 vs. 11.8 months, log rank *p* < 0.001, MV HR 3.07 [95% CI 1.69–5.60]), though had no significant correlation with ORR,^61^ suggesting a role in accelerating acquired resistance. Supporting this, post-sotorasib progression in the CodeBreak trial revealed emergent SMARCA4 mutations (15% allele frequency).^31^ Additionally, triple mutations involving KEAP1, SMARCA4, and CDKN2A were significantly associated with a reduced ORR.^61^

### Retinoblastoma protein

RB is a key regulator of the cell cycle, acting as a critical gatekeeper for the G1/S phase transition. Its function relies on its binding to E2F transcription factors, which can inhibit the transcription of genes necessary for S phase entry and keep cells in a quiescent state. The inhibition of ERK-regulated outputs, including E2F-mediated transcription, was revealed to be related to apoptosis induction in MRTX849-treated CDX models.^23^ A recent *in vitro* study demonstrated that a LOF mutation (D32fs) in RB triggered resistance against KRAS G12Ci in HCC44 cells. In KRAS G12C-mutated cells that developed resistance to AMG510, the phosphorylated form of R was less responsive to the inhibitor, indicating that RB inactivation could enable these cells to evade the growth-inhibitory effects of KRAS G12Ci.^129^

### Neurofibromatosis type 1

The NF1 gene encodes neurofibromin, a protein that is critical in regulating cell behaviors, particularly in modulating the RAS signaling pathway. As a GAP, NF1 can promote the conversion of RAS from a GTP-bound active state to a GDP-bound inactive state, thus inactivating RAS, which is essential for maintaining normal cellular signaling. For example, patients with NF1 A26465S (0.6%) at baseline experienced an 11% tumor reduction after 4 months of sotorasib treatment, and patients with SCLC with treatment-emerging NF1 N1004Ifs∗8 (0.3%) progressed with a 58% increase in tumor size, accompanied by MYC amplification (3.5-fold).^31^ Additionally, the LOF NF1 R2637∗ mutation was identified in a patient with appendiceal cancer as the sole treatment-emerging mutation in cfDNA who developed resistance to adagrasib. In another study related to the use of SOS1 inhibitor MRTX0902 in combination with KRAS G12Ci, mutations in NF1 could enhance cell survival under drug pressure through CRISPR/CAS9 screening analysis, indicating an undeniable role of LOF mutations of NF1 in the development of resistance.^38^

### Casitas B-lineage lymphoma

Casitas B-lineage lymphoma (CBL) can catalyze the direct transfer of ubiquitin from ubiquitin-conjugating enzyme (E2) to its protein substrates, typically RTKs, leading to their ubiquitination and subsequent degradation in the lysosome. In the presence of CBL mutations that affect activities of its E3 ubiquitin ligase, oncogenesis might be induced by the resultant hyperactivation of RTK-mediated pathways such as EGFR.^130^ CBL mutations on RING finger also exhibited oncogenic property by abnormally activating PI3K/AKT pathway due to the TCR-directed increased phosphorylation of CBL on Y731 which recruited the p85 regulatory subunit of PI3K,^131^ as well as increased interaction with LYN and PIK3 regulatory subunit1.^132^ SgRNAs targeting CBL, which encodes an RF class E3 ubiquitin-protein ligase involved in cell signaling and protein ubiquitination, were significantly enriched in a CRISPR/CAS9 screening performed in H2122 cells *in vitro.*^23^

### Leucine zipper-like transcription regulator 1

Leucine zipper-like transcription regulator 1 (LZTR1) is a gene that encodes the LZTR1 protein, a member of the BTB-kelch superfamily. LZTR1 functions as a regulator of both canonical and non-canonical RAS GTPases, facilitating the proteasomal degradation of various RAS proteins (i.e., NRAS, KRAS, HRAS, MRAS, and RIT1).^133^ Inactivation of LZTR1 resulted in reduced ubiquitination and increased localization of endogenous KRAS at the plasma membrane.^134^ Loss of LZTR1 would promote the occurrence of resistance to G12Ci, potentially through feedback reactivation of RAS signaling via WT NRAS and HRAS after KRAS G12C inhibition.^135^ So far, there is a need to continue to explore the implications of LZTR1 in RAS pathway regulation and therapeutic resistance.

### Scribble

The Scribble (SCRIB) gene encodes the Scribble protein, a scaffold protein crucial for maintaining cell polarity, regulating cell migration, and controlling cell proliferation in epithelial cells. SCRIB is often deregulated in human lung cancers.^136^ SCRIB can interact with SHOC2, preventing its dephosphorylation of CRAF at Ser259, and thus counteract the MRAS-mediated formation of the ternary complex with SHOC2 and PP1 that activates CRAF by dephosphorylating Ser259.^137^ Regarding KRAS, tumors in SCRIB^f/f^/KRAS^G12D^ mice exhibited greater invasiveness than those in KRAS^G12D^ mice, potentially related to the reduced or mislocalized E-cadherin expression, hyperactivation of the MAPK-ERK pathway, increased collagen deposition, and heightened macrophage infiltration.^136^ Mis-localization of the apical-basal polarity protein SCRIB, which activates the EMT program, has been identified as a non-genetic mechanism of adaptive resistance in the NCI-H358 and LU65 cell lines.^138^ KRAS G12Ci treatment led to suppressed KRAS, which further inhibited MAPK signaling, thereby triggering post-translational regulation and suppression of zinc finger DHHC-type containing palmitoyl S-acyltransferase ZDHHC7. This would induce SCRIB mis-localization, resulting in the activation of YAP signaling and its nuclear translocation. Subsequently, YAP could upregulate the expression of MRAS to stimulate a molecular switch from SCRIB-SHOC2 to the formation of the MRAS:SHOC2:PP1C complex, promoting the re-activation of MAPK signaling eventually.^138^

## Preclinical models for studying resistance to KRAS G12Ci

### Cell lines

**Figure 4:**
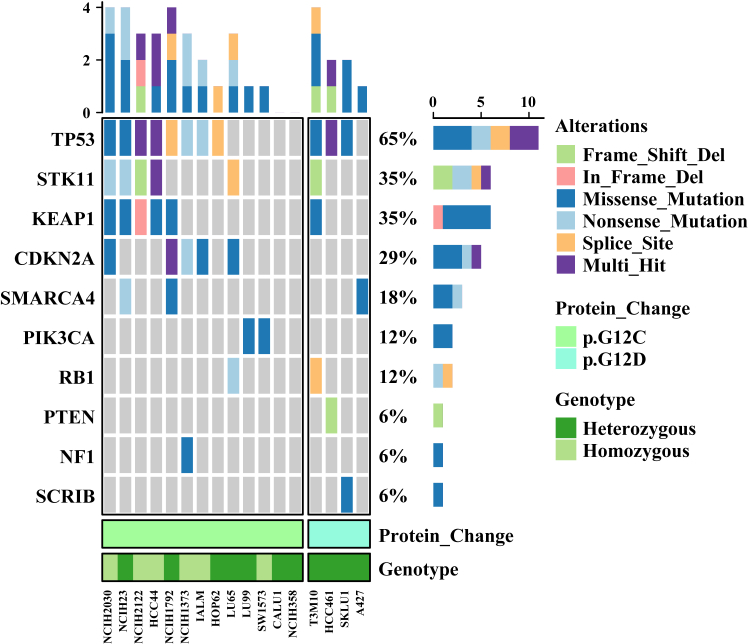
Co-mutation landscape of KRAS-mutant cell lines from DepMap This figure illustrates the co-mutation profiles of KRAS G12C- and G12D-mutant cancer cell lines based on data from the Cancer Dependency Map (DepMap) portal. Key co-mutated tumor suppressor genes include STK11, KEAP1, CDKN2A, SMARCA4, PIK3CA, RB1, PTEN, NF1, and SCRIB. Alteration types (e.g., frameshift deletions, missense mutations), protein changes (p.G12C and p.G12D), and genotypes (heterozygous/homozygous) are highlighted.

Indeed, there is a universal presence of KRAS G12C mutations across tumor cells. It should be acknowledged that heterogeneous co-mutation patterns and differential KRAS pathway dependency drive cell line-specific variations in drug sensitivity and resistance mechanisms (Figure 4). Typically, resistance in preclinical studies is modeled through gradual drug escalation to select for resistant subpopulations under sustained selective pressure, and CRISPR/Cas9 screening to systematically identify resistance-associated oncogenes or tumor suppressors.

However, there may be different drug sensitivities, even within the same cell line, owing to several confounding factors such as variations in experimental conditions and culture methods. For example, drug sensitivity might be reduced by growing cells in a 2D adherent environment.^18^ Additionally, the criteria used to assess drug sensitivity, such as cell growth inhibitory rate or phosphorylated ERK and AKT levels, can also result in discrepancies in classifying cells as sensitive, moderately sensitive, or resistant. For instance, the H2122 cell line has been categorized as both resistant and sensitive in different studies.^42^^,^^131^

Subclonal genetic architecture further complicates resistance mechanisms. For example, in EGFR-mutated LUAD, baseline clonal TP53 and RB1 co-mutations (occurring in ∼9% of cases) predisposed tumors to small cell transformation upon EGFR TKI therapy.^139^ Similarly, co-culture experiments with H358 cells harboring inducible NRAS Q61K demonstrated that minor subclonal populations (<5%) could diminish the therapeutic efficacy of sotorasib.^31^ These findings highlight the critical yet understudied role of subclonal dynamics in resistance, a challenge compounded by technical limitations in clinical detection and data interpretation. It may be feasible to clarify the specific mechanisms underlying TSG co-mutations shaping adaptive resistance by integrating such preclinical insights with clinical observations, such as the universal progression of initial responders and lack of OS benefit in sotorasib trials. In addition, systematic profiling of subclonal architectures and microenvironmental crosstalk in model systems may also benefit the unveiling of context-specific vulnerabilities for combinatorial targeting.

### Organoids

Traditional 2D cell cultures remain a cost-effective tool for rapid drug screening and intrinsic cancer vulnerability identification.^140^ However, and importantly, 3D models better recapitulate tissue architecture and cell-microenvironment interactions. Organoids are self-organized 3D tissue culture systems that can mimic organ-specific histology and function.^141^ Generally, they outperform 2D systems in aspects of the retention of tissue-specific differentiation, compatibility with gene editing, scalability for high-throughput studies, and reduced costs compared to xenografts.^142^ Tumor organoids are directly derived from patient-derived tumor samples, PDX models, or murine tumors, enabling biomarker discovery and drug response profiling, though their applications are restricted by their establishment success rates.^143^

For example, Tong et al. generated “plastic” (SCC morphology-positive) and “stable” (SCC-negative) organoids from KRAS^LSL−G12D/+^/LKB1^f/f^ (K_d_L) mice to study KRASG12D inhibitor (MRTX1133) resistance.^43^ Consequently, the former (i.e., plastic) organoids exhibiting SCC transcriptional signatures demonstrated intrinsic resistance, highlighting their utility for dissecting morphology-driven resistance mechanisms. Moreover, compared to xenografts, organoids allow for faster cultivation, easier manipulation (e.g., immunohistochemistry or multi-omics profiling), and longitudinal tracking of therapy-induced morphological shifts.

Emerging 3D systems also incorporate TME components. Hydrogel-based matrices were used to simulate ECM stiffness and composition to grow multicellular spheroids.^144^ In prostate cancer, synthetic hydrogels were found to mirror the role of ECM remodeling in neuroendocrine resistance to EZH2/DED inhibitors,^59^ identifying combinatorial strategies to overcome ECM-mediated drug tolerance. Similarly, organoid-immune co-cultures integrating patient-derived tumor cells, tumor-infiltrating lymphocytes, and PBMCs could elucidate the mechanism underlying immune-stromal crosstalk modulating KRAS G12Ci responses, though this remains under-explored in lung cancer.^145^

### Mouse models

Genetically engineered mouse models (GEMMs) of lung cancer facilitate a precise spatiotemporal control of oncogene activation and tumor suppressor inactivation, recapitulating critical aspects of tumor initiation, progression, and microenvironmental interactions.^146^ We also summarize key GEMMs used to study resistance mechanisms to KRAS G12Ci and their phenotypic outcomes (Table 1). For instance, adagrasib-resistant K_C_L mouse tumors displayed squamous pathology, while KRAS^LSL−G12C/+^/Trp53^f/f^ (K_CP_) retained ADC pathology.^46^ The KRAS^G12D^/LKB1^L/L^ (K_D_L) model is a well-established system for studying the mechanisms underlying spontaneous AST, progressing from atypical adenomatous hyperplasia through ADC and a mixed type defined as adenosquamous cell carcinoma (mAd-SCC) within 8–12 weeks post Ad-Cre induction.^56^^,^^64^ Building on this, Han X et al. demonstrated that SPC-CreERT-driven KRAS^G12D^/LKB1^f/f^ tumors originated from type II pneumocytes, recapitulating the mAd-SCC/SCC progression timeline (9–16 weeks).^70^ Meanwhile, by inter-crossing K_D_L and KRAS^LSL−G12C/+^ (KC) to generate K_C_L progeny, Mukhopadhyay S et al. developed MRTX849-resistant mouse models after 3–6 months of treatment.^66^ Concepcion et al. revealed SMARCA4-deficient tumors in KPS models predominantly arose from club cells, highlighting cell-of-origin dependencies in therapeutic resistance.^126^

**Table 1:** Summary of key phenotypes in GEMMs with KRAS mutations

GEMMs	Key Phenotypes	Relevance to NSCLC	Reference
KRAS^LSL−G12C/+^	ADCs	Mirror KRAS G21C-driven lung adenocarcinoma in patients	Li et al.^48^
KRAS^LSL−G12D/+^	ADCs	Mirror KRAS G21D-driven lung adenocarcinoma in patients	Jackson et al.^68^
KRAS^LSL−G12C/+^/LKB1^f/f^ (K_C_L)	Initial response to MRTX849 treatment (100 mg/kg/d), followed by resistant tumors (≥20 weeks) displaying squamous pathology (Ad-Cre 2 × 10^6^ PFU);	Recapitulates human KRAS/LKB1 co-mutant NSCLC showing squamous histology and resistance to G12Ci	Tong et al.^46^
	Complete response after 1-month MRTX849 treatment (100 mg/kg/d) with post-withdrawal recurrence; resistant tumors 3–6 months post Ad-Cre (1 × 10^7^ PFU)		Mukhopadhyay et al.^66^
KRAS^LSL−G12C/+^/Trp53^f/f^ (K_C_P)	Adagrasib-resistant tumors maintaining ADC pathology	Reflects resistance patterns in patients with KRAS/TP53-mutant NSCLC	Tong et al.^46^
KRAS^LSL−G12D/+^/LKB1^f/f^ (K_D_L)	Spontaneous AST	Mirrors AST observed in KRAS/LKB1-mutant lung cancer, dissects the relationship between KRAS inhibition and AST	Tong et al.^46^
SP-C^CreERT^/KRAS^G12D^/LKB1^f/f^	Development of SCC from type II pneumocytes	Models KRAS/LKB1-driven SCC in patients with NSCLC and identifies the cell of origin for SCC in this context	Tong et al.^46^
KRAS^LSL−G12D^/SMARCA4^f/f^	Shorter OS and lower tumor grade than KRAS^LSL−G12D^/SMARCA4^+/+^, along with decreased activation of AKT and MAPK, as well as high levels of senescence markers p16^INK4a^ and p21^Waf/Cip1^	Parallels poorer prognosis in KRAS/SMARCA4 co-mutant NSCLC	Malik et al.^125^
Kras^FrtStopFrt−G12D^/Spc^CreERT^/Smarca4^+/+^	Significant increase in lifespan compared to Kras^FrtStopFrt−G12D^/Spc^CreERT2^/SMARCA4^f/f^ (Smarca4 knockout after 3 weeks post-Adeno-FLp infection)	Identifies SMARCA4 as a potential specific vulnerability in KRAS-driven lung cancer	Malik et al.^125^
Kras^LSL−G12D^/p53^f/f^/Smarca4^f/f^ (KPS)or Kras^LSL−G12D^/p53^f/f^/Smarca4^f/+^(KPS-HET)	An increase in the fraction of early lesions (grades 1–2) and a decrease in more advanced grade 3 tumors in KPS mice compared with KP and KPS-HET animals; as well as the highest fraction of grade 4 lesions and metastases in KPS; Accelerated tumorigenesis in CCSP-driven KSP models, while restrained tumor progression in SPC-driven KSP models	Demonstrates the dual role of SMARCA4 in KRAS-derived lung tumor	Lissanu Deribe et al.^121^ Concepcion et al.^126^
Kras^LSL−G12D^/Scrib^fl/fl’^	Greater invasiveness than KRAS^LSL−G12D^ mice	Mirrors invasive progression in human KRAS/SCRIB co-mutant lung adenocarcinoma	Elsum et al.^136^

Furthermore, cell line-derived xenograft (CDX) models are widely used to assess the efficacy of therapeutic interventions, given their better mimicking effect of the TME dynamics and tumor-stroma interactions than conventional 2D cell culture. Such models are particularly useful for evaluating combination therapies that target resistance mechanisms in cell lines. However, their limitations should be taken with caution, such as cost-consuming and complicated processing, as well as limited genetic diversity due to their origin from specific cell lines. H358 and MIA PaCa-2 CDX models, with significant inhibitory effects on ERK and S6 phosphorylation, have been identified as MRTX849-responsive tumor models.^23^ However, no clear correlation was reported between drug response in cell lines and their corresponding xenograft models.^23^ Hallin et al. further found that genetic alterations (e.g., KEAP1, STK11, TP53, and CDKN2A) exhibited no predictive performance on MRTX849 response across various KRAS G12C CDX models.^23^ Patient-derived xenograft (PDX) models better preserve tumor heterogeneity and stromal composition. Their expansion in modeling acquired resistance mechanisms, however, is restricted by their long establishment time, reliance on immunocompromised mice, and the tendency for resistant clones to be outcompeted by non-resistant clones during expansion. Yulei Zhao et al. used a subcutaneous injection-derived PDX model to identify secondary KRAS (G13D) and BRAF (S147N) mutations as treatment-emerging alterations, which were concordant with findings from cell-based experiments and clinical data.^31^ Hallin et al. utilized a CRISPR/Cas9 screening, where sgRNAs targeting *KEAP1* were enriched in subcutaneous xenografts derived from MRTX849-treated H2122 cells.^23^ Some other studies also employed PDX models to validate the efficacy of KRAS G12Ci in combination with other drugs rather than to investigate resistance mechanisms.^147^

## Therapeutic strategies for tumor suppressor gene-deficient non-small cell lung cancer

The profound heterogeneity of tumor suppressor gene (TSG) alterations necessitates functional stratification to unlock precision oncology. While emerging evidence supports specific combinations, such as G12Ci with CDK4/6 or SHP2 inhibitors, the field remains in a developing phase where most therapeutic strategies are still being investigated as monotherapies targeting TSG-related vulnerabilities. This section systematically outlines these core therapeutic avenues, providing a framework for rational combination design as clinical evidence continues to evolve.

First, restoring tumor suppressor function through structural correctors or epigenetic modulators. APR-246 and PC14586 can bind tothe DNA binding region of mutant p53 and restore its conformation and transcriptional function, currently in clinical trials (NCT04383938, NCT04585750). Histone deacetylase inhibitors (vorinostat, panobinostat) are under investigation for NF1-associated tumors (NCT00251589, NCT06693284).

Second, targeting oncogenic dependencies from tumor suppressor loss. STK11/LKB1 loss increases sensitivity to metabolic stress inducers (e.g., metformin, tunicamycin).^52^ Strategies targeting KEAP1 inactivation focus on inhibiting NRF2 and downstream metabolic pathways. For example, the glutaminase inhibitor telaglenastat (CB-839) in Phase II trials for KEAP1/NFE2L2-mutant NSCLC (NCT04265534, NCT03872427).^91^ CDKN loss leads to CDK hyperactivation. The combination of MRTX849 and palbociclib, a CDK4/6 inhibitor, resulted in nearly complete inhibition of pRB in preclinical models.^23^ At present, several clinical trials involving CDK inhibitors for lung cancer are underway, including palbociclib (NCT04591431), BGB-43395 (NCT06120283), PF-07220060 (NCT04557449), and PF-07248144 (NCT04606446). Additionally, MEK-ERK, PI3K-AKT-mTOR, and Hippo pathway activation occur in various TSG-deficient contexts (NF1, STK11, Scribble, and PTEN) and are actionable combinational targets in preclinical models.^65^^,^^66^^,^^119^ The dual mTORC1/2 inhibitor sapanisertib is being examined in the advanced setting in NSCLC (NCT02417701 and NCT04250545). For CBL loss-driven RTK activation, potential combination therapies such as EGFR inhibitor Panitumumab plus Sotorasib are being evaluated in clinical trials (NCT05993455). RB1 loss induces hyperactive E2F transcription and dependence on epigenetic regulators. Corresponding inhibitors (e.g., LSD1 inhibitor GSK2879552, PRMT5 inhibitor Pemrametostat, DOT1L inhibitor EPZ5676) show efficacy in preclinical models.^148^ Furthermore, PARP inhibitors are effective in tumors with DNA repair defects caused by tumor suppressor gene loss. Cancers with RB1 or STK11 deficiencies show sensitivity to PARP inhibition.^52^^,^^149^ Clinical trials evaluating PARP inhibitors in RB1-deficient cancers (NCT03227042) are currently underway.

Third, leveraging synthetic lethality. SMARCA4-deficient tumors develop a synthetic lethal dependency on its paralog, SMARCA2. Targeting this vulnerability, the SMARCA2 degrader PRT3789 has entered clinical trials (NCT05639751). RB1-deficient tumors show hypersensitivity to Aurora kinase inhibitors, demonstrating efficacy in preclinical studies.^150^

Fourth, reprogramming the immune microenvironment. While STK11 or KEAP1 inactivation causes immunotherapy resistance by suppressing the STING pathway, combining STING agonists is emerging as a novel strategy to overcome this resistance.^76^^,^^101^ In NF1-deficient tumors, PD-1 antibodies have been shown to improve survival in melanoma and other cancer types.^151^^,^^152^

Fifth, targeting oncogenic splice variants. Aberrant splicing generates oncogenic isoforms (e.g., ΔN-LKB1 from STK11) with opposing functions. Developing specific inhibitors against these variants represents a promising direction for precision therapy.

## Conclusion

KRAS G12Ci can reshape the therapeutic landscape of KRAS-mutated NSCLC; the presence of co-occurring TSG mutations constitutes a formidable barrier to durable responses. Rather than bystanders, these co-mutations can actively reprogram cellular signaling, metabolic states, and immune landscapes, shaping intrinsic and acquired resistance to G12C-targeted therapy. Currently, experimental and clinical efforts have been devoted to elucidating the multifaceted roles of alterations in STK11, KEAP1, CDKN2A, TP53, and SMARCA4. However, it still remains elusive regarding a unified understanding of the intrinsic mechanisms of these TSGs orchestrating resistance.

Specific co-mutation patterns may identify subgroups more likely to benefit from particular combination therapies, according to current preclinical and real-world studies. However, corresponding clinical translation is hindered by the existing variability in model systems, incomplete functional annotation of mutations, and the heterogeneous nature of resistance mechanisms across tissues and genetic contexts. Furthermore, despite well-documented mechanisms such as STK11-driven immune evasion or KEAP1-mediated redox adaptation, further validation is still necessitated to get to the bottom of the role of SCRIB mislocalization or CBL-mediated bypass signaling, and so forth.

Crucially, many conclusions are drawn from retrospective analyses or *in vitro* models, both of which are insufficient to recapitulate tumor-immune and stromal interactions. There is an urgent need for prospective studies and functionally validated biomarkers to enable rational stratification of patients. Moreover, comprehensive integration of multi-omics, spatial profiling, and longitudinal monitoring of tumor evolution may also be a feasible solution for capturing the temporal dynamics of resistance.

Ultimately, in order to overcome the proposed resistance, our future directions may include the identification of actionable vulnerabilities within TSG-mutated subsets and an understanding of their interplay with the TME. Overall, the future of KRAS G12Ci therapy lies in personalized, context-aware intervention strategies, whether through synthetic lethal approaches, targeted modulation of metabolic or epigenetic pathways, or restoration of immune competence.

## Data and code availability

The data supporting Figure 4 are openly available in Dependency Map (DepMap) portal (DepMap: The Cancer Dependency Map Project at Broad Institute), including KRAS mutation status (G12C/G12D) from the KRAS gene characterization page (KRAS DepMap Gene Summary) and co-mutation profiles of TP53, STK11, KEAP1, CDKN2A, SMARCA4, PIK3CA, RB1, NF1, and SCRIB from the custom downloads section (Data | DepMap Portal).

## Acknowledgments

This study used the databases from DepMap and the authors acknowledge the efforts of the corresponding institutes. The interpretation and reporting of these data are the sole responsibility of the authors. The graphical abstract in this article was created with BioRender.com. The study was supported by the AI grant of Tongji Hospital, Tongji Medical College, Huazhong University of Science and Technology (No. AI2025B06); the Henan Provincial Key Scientific and Technological Project (No. 232102310044) and the Joint Construction Project of Henan Medical Science and Technology Research (No. LHGJ20240017) to Xiangbo Jia and Lei Xu respectively.

## Author contributions

Linsha Zhu: investigation, writing-original draft, and revision and editing. Xiangbo Jia: investigation and writing-revision and editing. Shu Peng: conceptualization, investigation, and writing-original draft and revision. Bo Ai: supervision and writing – review. Li Chen: writing-review and editing. Xiangning Fu and Lei Xu: writing-review. Hua Yan: supervision and writing – review.

## Declaration of interests

The authors declare no competing interests.

## References

[1] Chevallier M., Borgeaud M., Addeo A., Friedlaender A. (2021). Oncogenic driver mutations in non-small cell lung cancer: Past, present and future. World J. Clin. Oncol..

[2] Barlesi F., Mazieres J., Merlio J.-P., Debieuvre D., Mosser J., Lena H., Ouafik L., Besse B., Rouquette I., Westeel V. (2016). Routine molecular profiling of patients with advanced non-small-cell lung cancer: results of a 1-year nationwide programme of the French Cooperative Thoracic Intergroup (IFCT). Lancet Lond. Engl..

[3] Ricciuti B., Alessi J.V., Elkrief A., Wang X., Cortellini A., Li Y.Y., Vaz V.R., Gupta H., Pecci F., Barrichello A. (2022). Dissecting the clinicopathologic, genomic, and immunophenotypic correlates of KRASG12D-mutated non-small-cell lung cancer. Ann. Oncol..

[4] Salem M.E., El-Refai S.M., Sha W., Puccini A., Grothey A., George T.J., Hwang J.J., O’Neil B., Barrett A.S., Kadakia K.C. (2022). Landscape of KRASG12C, Associated Genomic Alterations, and Interrelation With Immuno-Oncology Biomarkers in KRAS-Mutated Cancers. JCO Precis. Oncol..

[5] Arbour K.C., Rizvi H., Plodkowski A.J., Hellmann M.D., Knezevic A., Heller G., Yu H.A., Ladanyi M., Kris M.G., Arcila M.E. (2021). Treatment Outcomes and Clinical Characteristics of Patients with KRAS-G12C-Mutant Non-Small Cell Lung Cancer. Clin. Cancer Res..

[6] Bourne H.R., Sanders D.A., McCormick F. (1990). The GTPase superfamily: a conserved switch for diverse cell functions. Nature.

[7] Huang L., Guo Z., Wang F., Fu L. (2021). KRAS mutation: from undruggable to druggable in cancer. Signal Transduct. Target. Ther..

[8] Spira A.I., Tu H., Aggarwal S., Hsu H., Carrigan G., Wang X., Ngarmchamnanrith G., Chia V., Gray J.E. (2021). A retrospective observational study of the natural history of advanced non-small-cell lung cancer in patients with KRAS p.G12C mutated or wild-type disease. Lung Cancer.

[9] Singh N., Temin S., Baker S., Blanchard E., Brahmer J.R., Celano P., Duma N., Ellis P.M., Elkins I.B., Haddad R.Y. (2022). Therapy for Stage IV Non-Small-Cell Lung Cancer With Driver Alterations: ASCO Living Guideline. J. Clin. Oncol..

[10] Califano R., Landi L., Cappuzzo F. (2012). Prognostic and predictive value of K-RAS mutations in non-small cell lung cancer. Drugs.

[11] Park S., Kim J.-Y., Lee S.-H., Suh B., Keam B., Kim T.M., Kim D.-W., Heo D.S. (2017). KRAS G12C mutation as a poor prognostic marker of pemetrexed treatment in non-small cell lung cancer. Korean J. Intern. Med..

[12] Lim T.K.H., Skoulidis F., Kerr K.M., Ahn M.-J., Kapp J.R., Soares F.A., Yatabe Y. (2023). 10 KRAS G12C in advanced NSCLC: Prevalence, co-mutations, and testing. Lung Cancer Amst. Neth..

[13] Hamarsheh S., Groß O., Brummer T., Zeiser R. (2020). Immune modulatory effects of oncogenic KRAS in cancer. Nat. Commun..

[14] Mok T.S.K., Wu Y.-L., Kudaba I., Kowalski D.M., Cho B.C., Turna H.Z., Castro G., Srimuninnimit V., Laktionov K.K., Bondarenko I. (2019). Pembrolizumab versus chemotherapy for previously untreated, PD-L1-expressing, locally advanced or metastatic non-small-cell lung cancer (KEYNOTE-042): a randomised, open-label, controlled, phase 3 trial. Lancet Lond. Engl..

[15] Cox A.D., Der C.J. (2002). Ras Family Signaling: Therapeutic Targeting. Cancer Biol. Ther..

[16] Stephen A.G., Esposito D., Bagni R.K., McCormick F. (2014). Dragging Ras Back in the Ring. Cancer Cell.

[17] Lokhandwala J., Smalley T.B., Tran T.H. (2024). Structural perspectives on recent breakthrough efforts toward direct drugging of RAS and acquired resistance. Front. Oncol..

[18] Patricelli M.P., Janes M.R., Li L.-S., Hansen R., Peters U., Kessler L.V., Chen Y., Kucharski J.M., Feng J., Ely T. (2016). Selective Inhibition of Oncogenic KRAS Output with Small Molecules Targeting the Inactive State. Cancer Discov..

[19] Cherfils J., Zeghouf M. (2013). Regulation of Small GTPases by GEFs, GAPs, and GDIs. Physiol. Rev..

[20] Hunter J.C., Manandhar A., Carrasco M.A., Gurbani D., Gondi S., Westover K.D. (2015). Biochemical and Structural Analysis of Common Cancer-Associated KRAS Mutations. Mol. Cancer Res..

[21] Khan I., Rhett J.M., O’Bryan J.P. (2020). Therapeutic targeting of RAS: New hope for drugging the “undruggable.”. Biochim. Biophys. Acta. Mol. Cell Res..

[22] Canon J., Rex K., Saiki A.Y., Mohr C., Cooke K., Bagal D., Gaida K., Holt T., Knutson C.G., Koppada N. (2019). The clinical KRAS(G12C) inhibitor AMG 510 drives anti-tumour immunity. Nature.

[23] Hallin J., Engstrom L.D., Hargis L., Calinisan A., Aranda R., Briere D.M., Sudhakar N., Bowcut V., Baer B.R., Ballard J.A. (2020). The KRASG12C Inhibitor MRTX849 Provides Insight toward Therapeutic Susceptibility of KRAS-Mutant Cancers in Mouse Models and Patients. Cancer Discov..

[24] Hitchen N., Williams S., Desai J. (2025). Recent advances in therapeutic targeting of the KRAS pathway in cancer. Pharmacol. Ther..

[25] Maemondo M., Inoue A., Kobayashi K., Sugawara S., Oizumi S., Isobe H., Gemma A., Harada M., Yoshizawa H., Kinoshita I. (2010). Gefitinib or chemotherapy for non-small-cell lung cancer with mutated EGFR. N. Engl. J. Med..

[26] Mitsudomi T., Morita S., Yatabe Y., Negoro S., Okamoto I., Tsurutani J., Seto T., Satouchi M., Tada H., Hirashima T. (2010). Gefitinib versus cisplatin plus docetaxel in patients with non-small-cell lung cancer harbouring mutations of the epidermal growth factor receptor (WJTOG3405): an open label, randomised phase 3 trial. Lancet Oncol..

[27] H Y., M S., T S., S M., Y Y., I O., J T., M S., T H., S A. (2019). Final overall survival results of WJTOG3405, a randomized phase III trial comparing gefitinib versus cisplatin with docetaxel as the first-line treatment for patients with stage IIIB/IV or postoperative recurrent EGFR mutation-positive non-small-cell lung cancer. Ann. Oncol. Off. J. Eur. Soc. Med. Oncol..

[28] Mok T.S., Wu Y.-L., Thongprasert S., Yang C.-H., Chu D.-T., Saijo N., Sunpaweravong P., Han B., Margono B., Ichinose Y. (2009). Gefitinib or carboplatin-paclitaxel in pulmonary adenocarcinoma. N. Engl. J. Med..

[29] F S., Jv H. (2019). Co-occurring genomic alterations in non-small-cell lung cancer biology and therapy. Nat. Rev. Cancer.

[30] Awad M.M., Liu S., Rybkin I.I., Arbour K.C., Dilly J., Zhu V.W., Johnson M.L., Heist R.S., Patil T., Riely G.J. (2021). Acquired Resistance to KRASG12C Inhibition in Cancer. N. Engl. J. Med..

[31] Zhao Y., Murciano-Goroff Y.R., Xue J.Y., Ang A., Lucas J., Mai T.T., Da Cruz Paula A.F., Saiki A.Y., Mohn D., Achanta P. (2021). Diverse alterations associated with resistance to KRAS(G12C) inhibition. Nature.

[32] Dy G.K., Govindan R., Velcheti V., Falchook G.S., Italiano A., Wolf J., Sacher A.G., Takahashi T., Ramalingam S.S., Dooms C. (2023). 11 Long-Term Outcomes and Molecular Correlates of Sotorasib Efficacy in Patients With Pretreated KRAS G12C-Mutated Non–Small-Cell Lung Cancer: 2-Year Analysis of CodeBreaK 100. J. Clin. Oncol..

[33] De Langen A.J., Johnson M.L., Mazieres J., Dingemans A.-M.C., Mountzios G., Pless M., Wolf J., Schuler M., Lena H., Skoulidis F. (2023). Sotorasib versus docetaxel for previously treated non-small-cell lung cancer with KRASG12C mutation: a randomised, open-label, phase 3 trial. Lancet.

[34] Jänne P.A., Riely G.J., Gadgeel S.M., Heist R.S., Ou S.-H.I., Pacheco J.M., Johnson M.L., Sabari J.K., Leventakos K., Yau E. (2022). Adagrasib in Non-Small-Cell Lung Cancer Harboring a KRASG12C Mutation. N. Engl. J. Med..

[35] Sacher A.G., Miller W.H., Patel M.R., Paz-Ares L., Santoro A., Ahn M.-J., Dziadziuszko R., Freres P., Luo J., Bowyer S. (2025). Single-Agent Divarasib in Patients With KRAS G12C-Positive Non-Small Cell Lung Cancer: Long-Term Follow-Up of a Phase I Study. J. Clin. Oncol..

[36] Singhal A., Li B.T., O’Reilly E.M. (2024). Targeting KRAS in cancer. Nat. Med..

[37] McCormick F. (2020). Sticking it to KRAS: Covalent Inhibitors Enter the Clinic. Cancer Cell.

[38] Sudhakar N., Yan L., Qiryaqos F., Engstrom L.D., Laguer J., Calinisan A., Hebbert A., Waters L., Moya K., Bowcut V. (2024). The SOS1 Inhibitor MRTX0902 Blocks KRAS Activation and Demonstrates Antitumor Activity in Cancers Dependent on KRAS Nucleotide Loading. Mol. Cancer Ther..

[39] Xue J.Y., Zhao Y., Aronowitz J., Mai T.T., Vides A., Qeriqi B., Kim D., Li C., De Stanchina E., Mazutis L. (2020). Rapid non-uniform adaptation to conformation-specific KRAS(G12C) inhibition. Nature.

[40] Ryan M.B., Fece de la Cruz F., Phat S., Myers D.T., Wong E., Shahzade H.A., Hong C.B., Corcoran R.B. (2020). Vertical Pathway Inhibition Overcomes Adaptive Feedback Resistance to KRASG12C Inhibition. Clin. Cancer Res. Off. J. Am. Assoc. Cancer Res..

[41] Sg W., Jy S. (2018). Management of acquired resistance to EGFR TKI-targeted therapy in advanced non-small cell lung cancer. Mol. Cancer.

[42] Strohbehn G.W., Sankar K., Qin A., Kalemkerian G.P. (2022). An evaluation of sotorasib for the treatment of patients with non-small cell lung cancer with KRASG12C mutations. Expert Opin. Pharmacother..

[43] Yu H.A., Arcila M.E., Rekhtman N., Sima C.S., Zakowski M.F., Pao W., Kris M.G., Miller V.A., Ladanyi M., Riely G.J. (2013). Analysis of tumor specimens at the time of acquired resistance to EGFR-TKI therapy in 155 patients with EGFR-mutant lung cancers. Clin. Cancer Res..

[44] Koga T., Suda K., Fujino T., Ohara S., Hamada A., Nishino M., Chiba M., Shimoji M., Takemoto T., Arita T. (2021). *KRAS* Secondary Mutations That Confer Acquired Resistance to KRAS G12C Inhibitors, Sotorasib and Adagrasib, and Overcoming Strategies: Insights From In Vitro Experiments. J. Thorac. Oncol..

[45] Riedl J.M., Fece de la Cruz F., Lin J.J., Parseghian C., Kim J.E., Matsubara H., Barnes H., Caughey B., Norden B.L., Morales-Giron A.A. (2025). Genomic landscape of clinically acquired resistance alterations in patients treated with KRASG12C inhibitors. Ann. Oncol..

[46] Tong X., Patel A.S., Kim E., Li H., Chen Y., Li S., Liu S., Dilly J., Kapner K.S., Zhang N. (2024). 6 Adeno-to-squamous transition drives resistance to KRAS inhibition in LKB1 mutant lung cancer. Cancer Cell.

[47] Singh A., Greninger P., Rhodes D., Koopman L., Violette S., Bardeesy N., Settleman J. (2009). A Gene Expression Signature Associated with “K-Ras Addiction” Reveals Regulators of EMT and Tumor Cell Survival. Cancer Cell.

[48] Li S., Liu S., Deng J., Akbay E.A., Hai J., Ambrogio C., Zhang L., Zhou F., Jenkins R.W., Adeegbe D.O. (2018). Assessing Therapeutic Efficacy of MEK Inhibition in a KRASG12C-Driven Mouse Model of Lung Cancer. Clin. Cancer Res..

[49] Guin S., Ru Y., Wynes M.W., Mishra R., Lu X., Owens C., Barn A.E., Vasu V.T., Hirsch F.R., Kern J.A., Theodorescu D. (2013). Contributions of KRAS and RAL in non-small-cell lung cancer growth and progression. J. Thorac. Oncol..

[50] Skoulidis F., Byers L.A., Diao L., Papadimitrakopoulou V.A., Tong P., Izzo J., Behrens C., Kadara H., Parra E.R., Canales J.R. (2015). Co-occurring genomic alterations define major subsets of KRAS-mutant lung adenocarcinoma with distinct biology, immune profiles, and therapeutic vulnerabilities. Cancer Discov..

[51] Shepherd F.A., Domerg C., Hainaut P., Jänne P.A., Pignon J.-P., Graziano S., Douillard J.-Y., Brambilla E., Le Chevalier T., Seymour L. (2013). Pooled analysis of the prognostic and predictive effects of KRAS mutation status and KRAS mutation subtype in early-stage resected non-small-cell lung cancer in four trials of adjuvant chemotherapy. J. Clin. Oncol..

[52] Trelford C.B., Shepherd T.G. (2025). Insights into targeting LKB1 in tumorigenesis. Genes Dis..

[53] Granado-Martínez P., Garcia-Ortega S., González-Sánchez E., McGrail K., Selgas R., Grueso J., Gil R., Naldaiz-Gastesi N., Rhodes A.C., Hernandez-Losa J. (2020). STK11 (LKB1) missense somatic mutant isoforms promote tumor growth, motility and inflammation. Commun. Biol..

[54] Sanchez-Vega F., Mina M., Armenia J., Chatila W.K., Luna A., La K.C., Dimitriadoy S., Liu D.L., Kantheti H.S., Saghafinia S. (2018). Oncogenic Signaling Pathways in The Cancer Genome Atlas. Cell.

[55] Judd J., Abdel Karim N., Khan H., Naqash A.R., Baca Y., Xiu J., VanderWalde A.M., Mamdani H., Raez L.E., Nagasaka M. (2021). Characterization of KRAS Mutation Subtypes in Non-small Cell Lung Cancer. Mol. Cancer Ther..

[56] Schabath M.B., Welsh E.A., Fulp W.J., Chen L., Teer J.K., Thompson Z.J., Engel B.E., Xie M., Berglund A.E., Creelan B.C. (2016). 9 Differential association of STK11 and TP53 with KRAS mutation-associated gene expression, proliferation and immune surveillance in lung adenocarcinoma. Oncogene.

[57] Kaufman J.M., Amann J.M., Park K., Arasada R.R., Li H., Shyr Y., Carbone D.P. (2014). *LKB1* Loss Induces Characteristic Patterns of Gene Expression in Human Tumors Associated with NRF2 Activation and Attenuation of PI3K-AKT. J. Thorac. Oncol..

[58] Ji H., Ramsey M.R., Hayes D.N., Fan C., McNamara K., Kozlowski P., Torrice C., Wu M.C., Shimamura T., Perera S.A. (2007). LKB1 modulates lung cancer differentiation and metastasis. Nature.

[59] Pons-Tostivint E., Lugat A., Fontenau J.-F., Denis M.G., Bennouna J. (2021). STK11/LKB1 Modulation of the Immune Response in Lung Cancer: From Biology to Therapeutic Impact. Cells.

[60] F S., Bt L., Gk D., Tj P., Gs F., J W., A I., M S., H B., F B. (2021). Sotorasib for Lung Cancers with KRAS p.G12C Mutation. N. Engl. J. Med..

[61] Negrao M.V., Araujo H.A., Lamberti G., Cooper A.J., Akhave N.S., Zhou T., Delasos L., Hicks J.K., Aldea M., Minuti G. (2023). 12 Co-mutations and KRAS G12C inhibitor efficacy in advanced NSCLC. Cancer Discov..

[62] Li A., Wang Y., Yu Z., Tan Z., He L., Fu S., Shi M., Du W., Luo L., Li Z. (2023). STK11/LKB1-Deficient Phenotype Rather Than Mutation Diminishes Immunotherapy Efficacy and Represents STING/Type I Interferon/CD8+ T-Cell Dysfunction in NSCLC. J. Thorac. Oncol..

[63] Borzi C., Galli G., Ganzinelli M., Signorelli D., Vernieri C., Garassino M.C., Sozzi G., Moro M. (2020). Beyond LKB1 Mutations in Non-Small Cell Lung Cancer: Defining LKB1less Phenotype to Optimize Patient Selection and Treatment. Pharm. Basel Switz..

[64] Gao Y., Xiao Q., Ma H., Li L., Liu J., Feng Y., Fang Z., Wu J., Han X., Zhang J. (2010). LKB1 inhibits lung cancer progression through lysyl oxidase and extracellular matrix remodeling. Proc. Natl. Acad. Sci. USA.

[65] Mohseni M., Sun J., Lau A., Curtis S., Goldsmith J., Fox V.L., Wei C., Frazier M., Samson O., Wong K.-K. (2014). A genetic screen identifies an LKB1–MARK signalling axis controlling the Hippo–YAP pathway. Nat. Cell Biol..

[66] Mukhopadhyay S., Huang H.-Y., Lin Z., Ranieri M., Li S., Sahu S., Liu Y., Ban Y., Guidry K., Hu H. (2023). 5 Genome-Wide CRISPR Screens Identify Multiple Synthetic Lethal Targets That Enhance KRASG12C Inhibitor Efficacy. Cancer Res..

[67] Lenahan S.M., Sarausky H.M., Deming P., Seward D.J. (2024). 4 STK11 loss leads to YAP1-mediated transcriptional activation in human KRAS-driven lung adenocarcinoma cell lines. Cancer Gene Ther..

[68] Jackson E.L., Willis N., Mercer K., Bronson R.T., Crowley D., Montoya R., Jacks T., Tuveson D.A. (2001). Analysis of lung tumor initiation and progression using conditional expression of oncogenic K-ras. Genes Dev..

[69] Farago A.F., Snyder E.L., Jacks T. (2012). SnapShot: Lung cancer models. Cell.

[70] Han X., Li F., Fang Z., Gao Y., Li F., Fang R., Yao S., Sun Y., Li L., Zhang W. (2014). 1 Transdifferentiation of lung adenocarcinoma in mice with Lkb1 deficiency to squamous cell carcinoma. Nat. Commun..

[71] Zhao B., Li L., Wang L., Wang C.-Y., Yu J., Guan K.-L. (2012). Cell detachment activates the Hippo pathway via cytoskeleton reorganization to induce anoikis. Genes Dev..

[72] Gao Y., Zhang W., Han X., Li F., Wang X., Wang R., Fang Z., Tong X., Yao S., Li F. (2014). 8 YAP inhibits squamous transdifferentiation of Lkb1-deficient lung adenocarcinoma through ZEB2-dependent DNp63 repression. Nat. Commun..

[73] Arbour K.C., Jordan E., Kim H.R., Dienstag J., Yu H.A., Sanchez-Vega F., Lito P., Berger M., Solit D.B., Hellmann M. (2018). Effects of Co-occurring Genomic Alterations on Outcomes in Patients with KRAS-Mutant Non-Small Cell Lung Cancer. Clin. Cancer Res..

[74] Tsai Y.S., Woodcock M.G., Azam S.H., Thorne L.B., Kanchi K.L., Parker J.S., Vincent B.G., Pecot C.V. (2022). Rapid idiosyncratic mechanisms of clinical resistance to KRAS G12C inhibition. J. Clin. Investig..

[75] Qian Y., Galan-Cobo A., Guijarro I., Dang M., Molkentine D., Poteete A., Zhang F., Wang Q., Wang J., Parra E. (2023). MCT4-dependent lactate secretion suppresses antitumor immunity in LKB1-deficient lung adenocarcinoma. Cancer Cell.

[76] Della Corte C.M., Byers L.A. (2019). Evading the STING: LKB1 Loss Leads to STING Silencing and Immune Escape in KRAS-Mutant Lung Cancers. Cancer Discov..

[77] Kitajima S., Ivanova E., Guo S., Yoshida R., Campisi M., Sundararaman S.K., Tange S., Mitsuishi Y., Thai T.C., Masuda S. (2019). Suppression of STING Associated with LKB1 Loss in KRAS-Driven Lung Cancer. Cancer Discov..

[78] Ricciuti B., Arbour K.C., Lin J.J., Vajdi A., Vokes N., Hong L., Zhang J., Tolstorukov M.Y., Li Y.Y., Spurr L.F. (2022). Diminished Efficacy of Programmed Death-(Ligand)1 Inhibition in *STK11*- and *KEAP1*-Mutant Lung Adenocarcinoma Is Affected by *KRAS* Mutation Status. J. Thorac. Oncol..

[79] Koyama S., Akbay E.A., Li Y.Y., Aref A.R., Skoulidis F., Herter-Sprie G.S., Buczkowski K.A., Liu Y., Awad M.M., Denning W.L. (2016). STK11/LKB1 Deficiency Promotes Neutrophil Recruitment and Proinflammatory Cytokine Production to Suppress T-cell Activity in the Lung Tumor Microenvironment. Cancer Res..

[80] Kwak M.-K., Kensler T.W. (2010). Targeting NRF2 signaling for cancer chemoprevention. Toxicol. Appl. Pharmacol..

[81] Singh A., Misra V., Thimmulappa R.K., Lee H., Ames S., Hoque M.O., Herman J.G., Baylin S.B., Sidransky D., Gabrielson E. (2006). Dysfunctional KEAP1-NRF2 interaction in non-small-cell lung cancer. PLoS Med..

[82] Romero R., Sayin V.I., Davidson S.M., Bauer M.R., Singh S.X., LeBoeuf S.E., Karakousi T.R., Ellis D.C., Bhutkar A., Sánchez-Rivera F.J. (2017). Keap1 loss promotes Kras-driven lung cancer and results in dependence on glutaminolysis. Nat. Med..

[83] Arbour K.C., Jordan E., Kim H.R., Dienstag J., Yu H.A., Sanchez-Vega F., Lito P., Berger M., Solit D.B., Hellmann M. (2018). Effects of Co-occurring Genomic Alterations on Outcomes in Patients with KRAS-Mutant Non–Small Cell Lung Cancer. Clin. Cancer Res..

[84] Julian C., Pal N., Gershon A., Evangelista M., Purkey H., Lambert P., Shi Z., Zhang Q. (2023). Overall survival in patients with advanced non-small cell lung cancer with KRAS G12C mutation with or without STK11 and/or KEAP1 mutations in a real-world setting. BMC Cancer.

[85] Knetki-Wróblewska M., Wojas-Krawczyk K., Krawczyk P., Krzakowski M. (2024). Emerging insights into STK11, KEAP1 and KRAS mutations: implications for immunotherapy in patients with advanced non-small cell lung cancer. Transl. Lung Cancer Res..

[86] Scheffler M., Ihle M.A., Hein R., Merkelbach-Bruse S., Scheel A.H., Siemanowski J., Brägelmann J., Kron A., Abedpour N., Ueckeroth F. (2019). K-ras Mutation Subtypes in NSCLC and Associated Co-occuring Mutations in Other Oncogenic Pathways. J. Thorac. Oncol..

[87] Shang X., Li Z., Sun J., Zhao C., Lin J., Wang H. (2021). Survival analysis for non-squamous NSCLC patients harbored STK11 or KEAP1 mutation receiving atezolizumab. Lung Cancer.

[88] Galan-Cobo A., Sitthideatphaiboon P., Qu X., Poteete A., Pisegna M.A., Tong P., Chen P.-H., Boroughs L.K., Rodriguez M.L.M., Zhang W. (2019). LKB1 and KEAP1/NRF2 Pathways Cooperatively Promote Metabolic Reprogramming with Enhanced Glutamine Dependence in KRAS-Mutant Lung Adenocarcinoma. Cancer Res..

[89] Li F., Han X., Li F., Wang R., Wang H., Gao Y., Wang X., Fang Z., Zhang W., Yao S. (2015). LKB1 Inactivation Elicits a Redox Imbalance to Modulate Non-small Cell Lung Cancer Plasticity and Therapeutic Response. Cancer Cell.

[90] Jeon S.-M., Chandel N.S., Hay N. (2012). AMPK regulates NADPH homeostasis to promote tumour cell survival during energy stress. Nature.

[91] Xu K., Ma J., Hall S.R.R., Peng R.-W., Yang H., Yao F. (2023). Battles against aberrant KEAP1-NRF2 signaling in lung cancer: intertwined metabolic and immune networks. Theranostics.

[92] Heiss E.H., Schachner D., Zimmermann K., Dirsch V.M. (2013). Glucose availability is a decisive factor for Nrf2-mediated gene expression. Redox Biol..

[93] Wohlhieter C.A., Richards A.L., Uddin F., Hulton C.H., Quintanal-Villalonga À., Martin A., de Stanchina E., Bhanot U., Asher M., Shah N.S. (2020). Concurrent Mutations in STK11 and KEAP1 Promote Ferroptosis Protection and SCD1 Dependence in Lung Cancer. Cell Rep..

[94] Li C., Dong X., Du W., Shi X., Chen K., Zhang W., Gao M. (2020). LKB1-AMPK axis negatively regulates ferroptosis by inhibiting fatty acid synthesis. Signal Transduct. Target. Ther..

[95] Ying H., Kimmelman A.C., Lyssiotis C.A., Hua S., Chu G.C., Fletcher-Sananikone E., Locasale J.W., Son J., Zhang H., Coloff J.L. (2012). Oncogenic Kras maintains pancreatic tumors through regulation of anabolic glucose metabolism. Cell.

[96] Stratmann J.A., Althoff F.C., Doebel P., Rauh J., Trummer A., Hünerlitürkoglu A.N., Frost N., Yildirim H., Christopoulos P., Burkhard O. (2024). Sotorasib in KRAS G12C-mutated non-small cell lung cancer: A multicenter real-world experience from the compassionate use program in Germany. Eur. J. Cancer.

[97] Thummalapalli R., Bernstein E., Herzberg B., Li B.T., Iqbal A., Preeshagul I., Santini F.C., Eng J., Ladanyi M., Yang S.-R. (2023). Clinical and Genomic Features of Response and Toxicity to Sotorasib in a Real-World Cohort of Patients With Advanced KRAS G12C-Mutant Non-Small Cell Lung Cancer. JCO Precis. Oncol..

[98] Sreter K.B., Catarata M.J., von Laffert M., Frille A. (2024). Resistance to KRAS inhibition in advanced non-small cell lung cancer. Front. Oncol..

[99] Peindl M., Göttlich C., Crouch S., Hoff N., Lüttgens T., Schmitt F., Pereira J.G.N., May C., Schliermann A., Kronenthaler C. (2022). EMT, Stemness, and Drug Resistance in Biological Context: A 3D Tumor Tissue/In Silico Platform for Analysis of Combinatorial Treatment in NSCLC with Aggressive KRAS-Biomarker Signatures. Cancers.

[100] Best S.A., De Souza D.P., Kersbergen A., Policheni A.N., Dayalan S., Tull D., Rathi V., Gray D.H., Ritchie M.E., McConville M.J., Sutherland K.D. (2018). Synergy between the KEAP1/NRF2 and PI3K Pathways Drives Non-Small-Cell Lung Cancer with an Altered Immune Microenvironment. Cell Metab..

[101] Olagnier D., Brandtoft A.M., Gunderstofte C., Villadsen N.L., Krapp C., Thielke A.L., Laustsen A., Peri S., Hansen A.L., Bonefeld L. (2018). Nrf2 negatively regulates STING indicating a link between antiviral sensing and metabolic reprogramming. Nat. Commun..

[102] Serrano M., Hannon G.J., Beach D. (1993). A new regulatory motif in cell-cycle control causing specific inhibition of cyclin D/CDK4. Nature.

[103] Weinberg R.A. (1995). The retinoblastoma protein and cell cycle control. Cell.

[104] Yuan T.L., Amzallag A., Bagni R., Yi M., Afghani S., Burgan W., Fer N., Strathern L.A., Powell K., Smith B. (2018). Differential Effector Engagement by Oncogenic KRAS. Cell Rep..

[105] Zhao R., Choi B.Y., Lee M.-H., Bode A.M., Dong Z. (2016). Implications of Genetic and Epigenetic Alterations of CDKN2A (p16INK4a) in Cancer. EBioMedicine.

[106] Guan R.J., Fu Y., Holt P.R., Pardee A.B. (1999). Association of K-ras mutations with p16 methylation in human colon cancer. Gastroenterology.

[107] Tam K.W., Zhang W., Soh J., Stastny V., Chen M., Sun H., Thu K., Rios J.J., Yang C., Marconett C.N. (2013). *CDKN2A/p16* Inactivation Mechanisms and Their Relationship to Smoke Exposure and Molecular Features in Non–Small-Cell Lung Cancer. J. Thorac. Oncol..

[108] Aylon Y., Oren M. (2007). Living with p53, dying of p53. Cell.

[109] Janic A., Valente L.J., Wakefield M.J., Di Stefano L., Milla L., Wilcox S., Yang H., Tai L., Vandenberg C.J., Kueh A.J. (2018). DNA repair processes are critical mediators of p53-dependent tumor suppression. Nat. Med..

[110] Subramanian J., Govindan R. (2008). Molecular genetics of lung cancer in people who have never smoked. Lancet Oncol..

[111] Bouaoun L., Sonkin D., Ardin M., Hollstein M., Byrnes G., Zavadil J., Olivier M. (2016). TP53 Variations in Human Cancers: New Lessons from the IARC TP53 Database and Genomics Data. Hum. Mutat..

[112] Bj A., A J., Y C., C C., Ec L., St D., Aj K., Jp B., G D., La O. (2018). Mutant TRP53 exerts a target gene-selective dominant-negative effect to drive tumor development. Genes Dev..

[113] Wang Z., Burigotto M., Ghetti S., Vaillant F., Tan T., Capaldo B.D., Palmieri M., Hirokawa Y., Tai L., Simpson D.S. (2024). Loss-of-Function but Not Gain-of-Function Properties of Mutant TP53 Are Critical for the Proliferation, Survival, and Metastasis of a Broad Range of Cancer Cells. Cancer Discov..

[114] Molina-Vila M.A., Bertran-Alamillo J., Gascó A., Mayo-de-las-Casas C., Sánchez-Ronco M., Pujantell-Pastor L., Bonanno L., Favaretto A.G., Cardona A.F., Vergnenègre A. (2014). Nondisruptive p53 mutations are associated with shorter survival in patients with advanced non-small cell lung cancer. Clin. Cancer Res. Off. J. Am. Assoc. Cancer Res..

[115] Lee J.-W., Kim D.-M., Jang J.-W., Park T.-G., Song S.-H., Lee Y.-S., Chi X.-Z., Park I.Y., Hyun J.-W., Ito Y., Bae S.C. (2019). RUNX3 regulates cell cycle-dependent chromatin dynamics by functioning as a pioneer factor of the restriction-point. Nat. Commun..

[116] Lee Y.-S., Lee J.-W., Jang J.-W., Chi X.-Z., Kim J.-H., Li Y.-H., Kim M.-K., Kim D.-M., Choi B.-S., Kim E.-G. (2013). Runx3 Inactivation Is a Crucial Early Event in the Development of Lung Adenocarcinoma. Cancer Cell.

[117] Lee Y.-S., Bae S.-C. (2016). How do K-RAS-activated cells evade cellular defense mechanisms?. Oncogene.

[118] Molinari F., Frattini M. (2013). Functions and Regulation of the PTEN Gene in Colorectal Cancer. Front. Oncol..

[119] Chan C.-H., Chiou L.-W., Lee T.-Y., Liu Y.-R., Hsieh T.-H., Yang C.-Y., Jeng Y.-M. (2023). PAK and PI3K pathway activation confers resistance to KRASG12C inhibitor sotorasib. Br. J. Cancer.

[120] Lee J.Y., Bhandare R.R., Boddu S.H.S., Shaik A.B., Saktivel L.P., Gupta G., Negi P., Barakat M., Singh S.K., Dua K., Chellappan D.K. (2024). Molecular mechanisms underlying the regulation of tumour suppressor genes in lung cancer. Biomed. Pharmacother..

[121] Lissanu Deribe Y., Sun Y., Terranova C., Khan F., Martinez-Ledesma J., Gay J., Gao G., Mullinax R.A., Khor T., Feng N. (2018). Mutations in the SWI/SNF complex induce a targetable dependence on oxidative phosphorylation in lung cancer. Nat. Med..

[122] Nambirajan A., Jain D. (2021). Recent updates in thoracic SMARCA4-deficient undifferentiated tumor. Semin. Diagn. Pathol..

[123] Manolakos P., Boccuto L., Ivankovic D.S. (2024). A Critical Review of the Impact of SMARCA4 Mutations on Survival Outcomes in Non-Small Cell Lung Cancer. J. Pers. Med..

[124] Shaykevich A., Silverman I., Bandyopadhyaya G., Maitra R. (2023). BRG1: Promoter or Suppressor of Cancer? The Outcome of BRG1’s Interaction with Specific Cellular Pathways. Int. J. Mol. Sci..

[125] Malik S., Oshima M., Roy N., Kaushik S., Kuvshinova O., Wu W., Greer J.E., Green S., McMahon M., Jen K.-Y. (2020). SMARCA4 supports the oncogenic landscape of KRAS-driven lung tumors. bioRxiv.

[126] Concepcion C.P., Ma S., LaFave L.M., Bhutkar A., Liu M., DeAngelo L.P., Kim J.Y., Del Priore I., Schoenfeld A.J., Miller M. (2022). Smarca4 Inactivation Promotes Lineage-Specific Transformation and Early Metastatic Features in the Lung. Cancer Discov..

[127] Hodges H.C., Stanton B.Z., Cermakova K., Chang C.-Y., Miller E.L., Kirkland J.G., Ku W.L., Veverka V., Zhao K., Crabtree G.R. (2018). Dominant-negative SMARCA4 mutants alter the accessibility landscape of tissue-unrestricted enhancers. Nat. Struct. Mol. Biol..

[128] Clapier C.R., Verma N., Parnell T.J., Cairns B.R. (2020). Cancer-Associated Gain-of-Function Mutations Activate a SWI/SNF-Family Regulatory Hub. Mol. Cell.

[129] Qi W.L., Li H.Y., Wang Y., Xu L., Deng J.T., Zhang X., Wang Y.X., Meng L.H. (2023). Targeting PI3Kα overcomes resistance to KRasG12C inhibitors mediated by activation of EGFR and/or IGF1R. Acta Pharmacol. Sin..

[130] Yokouchi M., Kondo T., Houghton A., Bartkiewicz M., Horne W.C., Zhang H., Yoshimura A., Baron R. (1999). Ligand-induced Ubiquitination of the Epidermal Growth Factor Receptor Involves the Interaction of the c-Cbl RING Finger and UbcH7. J. Biol. Chem..

[131] Thien C.B.F., Blystad F.D., Zhan Y., Lew A.M., Voigt V., Andoniou C.E., Langdon W.Y. (2005). Loss of c-Cbl RING finger function results in high-intensity TCR signaling and thymic deletion. EMBO J..

[132] Belizaire R., Koochaki S.H.J., Udeshi N.D., Vedder A., Sun L., Svinkina T., Hartigan C., McConkey M., Kovalcik V., Bizuayehu A. (2021). CBL mutations drive PI3K/AKT signaling via increased interaction with LYN and PIK3R1. Blood.

[133] Chen S., Vedula R.S., Cuevas-Navarro A., Lu B., Hogg S.J., Wang E., Benbarche S., Knorr K., Kim W.J., Stanley R.F. (2022). Impaired Proteolysis of Noncanonical RAS Proteins Drives Clonal Hematopoietic Transformation. Cancer Discov..

[134] Bigenzahn J.W., Collu G.M., Kartnig F., Pieraks M., Vladimer G.I., Heinz L.X., Sedlyarov V., Schischlik F., Fauster A., Rebsamen M. (2018). LZTR1 is a regulator of RAS ubiquitination and signaling. Science.

[135] Rosell R., Jain A., Codony-Servat J., Jantus-Lewintre E., Morrison B., Ginesta J.B., González-Cao M. (2023). Biological insights in non-small cell lung cancer. Cancer Biol. Med..

[136] Elsum I.A., Yates L.L., Pearson H.B., Phesse T.J., Long F., O’Donoghue R., Ernst M., Cullinane C., Humbert P.O. (2014). Scrib heterozygosity predisposes to lung cancer and cooperates with KRas hyperactivation to accelerate lung cancer progression *in vivo*. Oncogene.

[137] Young L.C., Hartig N., Boned del Río I., Sari S., Ringham-Terry B., Wainwright J.R., Jones G.G., McCormick F., Rodriguez-Viciana P. (2018). SHOC2–MRAS–PP1 complex positively regulates RAF activity and contributes to Noonan syndrome pathogenesis. Proc. Natl. Acad. Sci. USA.

[138] Adachi Y., Kimura R., Hirade K., Yanase S., Nishioka Y., Kasuga N., Yamaguchi R., Ebi H. (2023). Scribble mis-localization induces adaptive resistance to KRAS G12C inhibitors through feedback activation of MAPK signaling mediated by YAP-induced MRAS. Nat. Cancer.

[139] Lee J.-K., Lee J., Kim S., Kim S., Youk J., Park S., An Y., Keam B., Kim D.-W., Heo D.S. (2017). Clonal History and Genetic Predictors of Transformation Into Small-Cell Carcinomas From Lung Adenocarcinomas. J. Clin. Oncol..

[140] Gazdar A.F., Girard L., Lockwood W.W., Lam W.L., Minna J.D. (2010). Lung Cancer Cell Lines as Tools for Biomedical Discovery and Research. J. Natl. Cancer Inst..

[141] Fang Z., Li P., Du F., Shang L., Li L. (2023). The role of organoids in cancer research. Exp. Hematol. Oncol..

[142] Li H., Chen Z., Chen N., Fan Y., Xu Y., Xu X. (2023). Applications of lung cancer organoids in precision medicine: from bench to bedside. Cell Commun. Signal..

[143] Dijkstra K.K., Monkhorst K., Schipper L.J., Hartemink K.J., Smit E.F., Kaing S., De Groot R., Wolkers M.C., Clevers H., Cuppen E., Voest E.E. (2020). Challenges in Establishing Pure Lung Cancer Organoids Limit Their Utility for Personalized Medicine. Cell Rep..

[144] Li Y., Kumacheva E. (2018). Hydrogel microenvironments for cancer spheroid growth and drug screening. Sci. Adv..

[145] Magré L., Verstegen M.M.A., Buschow S., van der Laan L.J.W., Peppelenbosch M., Desai J. (2023). Emerging organoid-immune co-culture models for cancer research: from oncoimmunology to personalized immunotherapies. J. Immunother. Cancer.

[146] de Seranno S., Meuwissen R. (2010). Progress and applications of mouse models for human lung cancer. Eur. Respir. J..

[147] Kazi A., Vasiyani H., Ghosh D., Bandyopadhyay D., Shah R.D., Vudatha V., Trevino J., Sebti S.M. (2025). FGTI-2734 Inhibits ERK Reactivation to Overcome Sotorasib Resistance in KRAS G12C Lung Cancer. J. Thorac. Oncol..

[148] Huang M.-F., Wang Y.-X., Chou Y.-T., Lee D.-F. (2024). Therapeutic Strategies for RB1-Deficient Cancers: Intersecting Gene Regulation and Targeted Therapy. Cancers.

[149] Zoumpoulidou G., Alvarez-Mendoza C., Mancusi C., Ahmed R.-M., Denman M., Steele C.D., Tarabichi M., Roy E., Davies L.R., Manji J. (2021). Therapeutic vulnerability to PARP1,2 inhibition in RB1-mutant osteosarcoma. Nat. Commun..

[150] Gong X., Du J., Parsons S.H., Merzoug F.F., Webster Y., Iversen P.W., Chio L.-C., Van Horn R.D., Lin X., Blosser W. (2019). Aurora A Kinase Inhibition Is Synthetic Lethal with Loss of the RB1 Tumor Suppressor Gene. Cancer Discov..

[151] Dai J., Bai X., Gao X., Tang L., Chen Y., Sun L., Wei X., Li C., Qi Z., Kong Y. (2023). Molecular underpinnings of exceptional response in primary malignant melanoma of the esophagus to anti-PD-1 monotherapy. J. Immunother. Cancer.

[152] Ascierto M.L., Makohon-Moore A., Lipson E.J., Taube J.M., McMiller T.L., Berger A.E., Fan J., Kaunitz G.J., Cottrell T.R., Kohutek Z.A. (2017). Transcriptional Mechanisms of Resistance to Anti-PD-1 Therapy. Clin. Cancer Res..

